# Supplementary material for: Localization of adaptive variants in human genomes using averaged one-dependence estimation
Source: Nat Commun. 2018 Feb 19;9:703. doi: 10.1038/s41467-018-03100-7 (PMC5818606; doi:10.1038/s41467-018-03100-7)
Supplement: Supplementary file 1 — Supplementary Information [file 41467_2018_3100_MOESM1_ESM.pdf]

Localization of adaptive variants in human genomes using averaged one-dependence estimation

Sugden *et al.*

Supplementary Figure 1

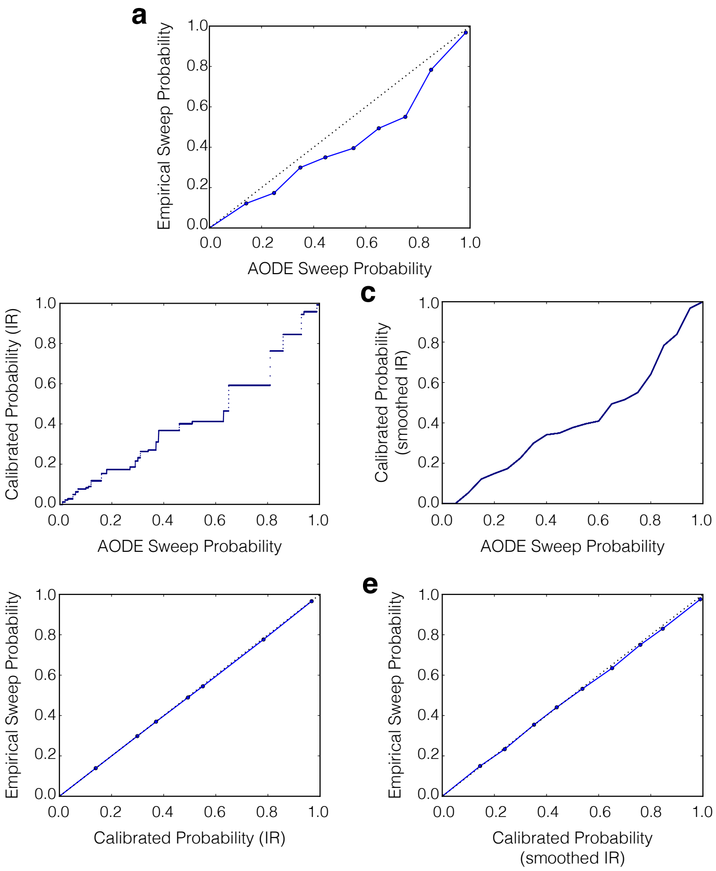

Calibration of SWIF(r) for analysis of data from the 1000 Genomes. For sweep site localization in data from the 1000 Genomes Project, we calibrated SWIF(r) based on a training dataset made up of 99.99% simulated neutral variants and 0.01% simulated sweep variants. We restricted the simulated sweep variants to those with present-day allele frequencies over 50%, since we have the most power in this realm, and wanted to avoid overcorrection of strong signals. **a)** Reliability curve[1] for uncalibrated SWIF(r) posterior probabilities using 10 evenly-spaced bins between 0 and 1; the x-axis plots the mean posterior probability within each bin, and the y-axis plots the fraction of sites within the bin that are sweep variants ("empirical sweep probability"). Uncalibrated, the probabilities that SWIF(r) calculates are slightly inflated. **b)** Isotonic regression (IR) mapping of probabilities in each bin to their corresponding empirical sweep probabilities, based on panel a. **c)** Our smoothed IR map, learned by interpolating between the midpoints of the piecewise constant segments inferred by isotonic regression in panel b (see also Supplementary Figure 3). **d)** Reliability curve for sweep probabilities calibrated with isotonic regression from panel b. **e)** Reliability curve for sweep probabilities calibrated with smoothed isotonic regression from panel c.

Supplementary Figure 2

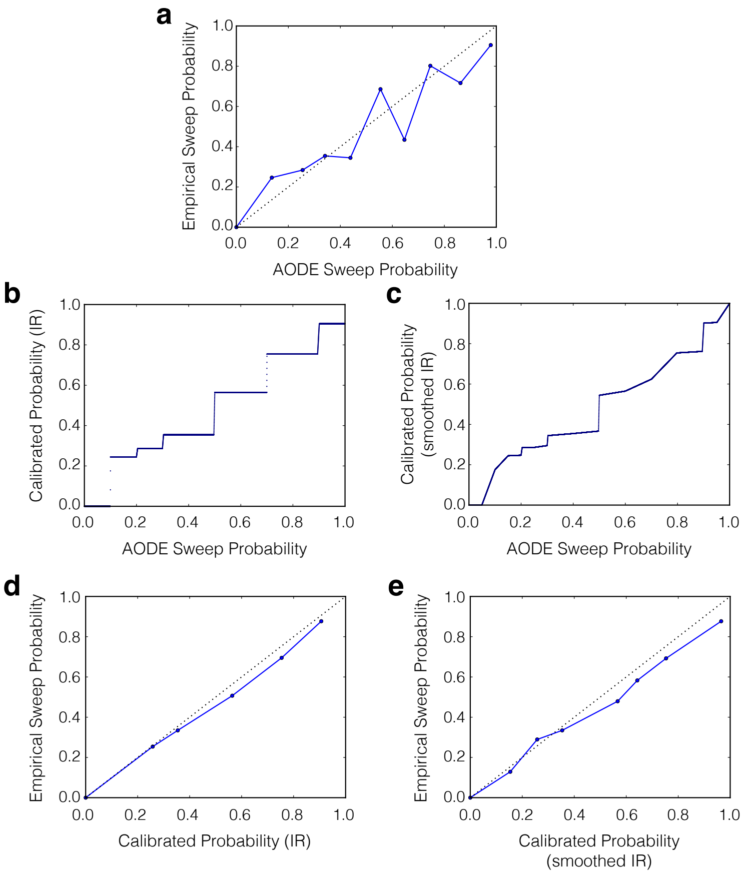

Calibration of SWIF(r) for analysis of SNP array data from the ‡Khomani San. For sweep site localization in array data from the ‡Khomani San, we calibrated SWIF(r) based on a training dataset made up of 99.95% simulated neutral variants and 0.05% simulated sweep variants with present-day allele frequencies over 50%. The slightly larger fraction of sweep simulations relative to the 1000 Genomes calibration is to allow for more sensitivity to older sweeps, and to account for the sparser SNP density of this dataset compared to the 1000 Genomes (phase 1). **a)** Reliability curve[1] for uncalibrated SWIF(r) posterior probabilities using 10 evenly-spaced bins between 0 and 1; the x-axis plots the mean posterior probability within each bin, and the y-axis plots the fraction of sites within the bin that are sweep variants ("empirical sweep probability"). **b)** Isotonic regression (IR) mapping of probabilities in each bin to their corresponding empirical sweep probabilities, from panel a. **c)** Our smoothed isotonic regression map, learned by interpolating between the midpoints of the piecewise constant segments inferred by isotonic regression in panel b (see also Supplementary Figure 3). **d)** Reliability curve for sweep probabilities calibrated with isotonic regression from panel b. **e)** Reliability curve for sweep probabilities calibrated with smoothed isotonic regression from panel c.

Supplementary Figure 3

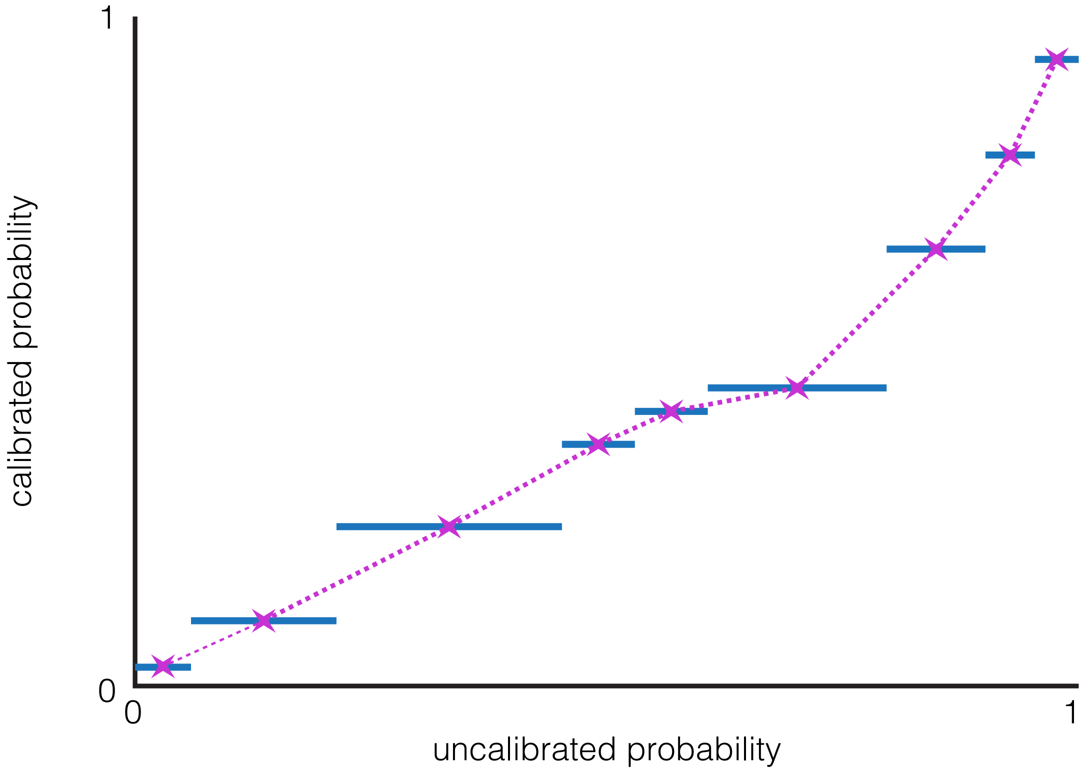

Schematic of smoothed isotonic regression for probability calibration. There are a few techniques for calibrating probabilities returned by a classifier so that of all of the data points that are given a $k\%$ probability of belonging to class A by the classifier, $k\%$ of those are indeed drawn from class A. Isotonic regression is a popular method because it makes no assumptions about the mapping function beyond requiring that it be monotonically increasing[2]. A downside, however, is that by nature, isotonic regression maps a range of input values to the same output value, which removes some information about which probabilities were larger than others. We implemented a "smoothed" isotonic regression for calibration that takes the piecewise constant mapping produced by isotonic calibration (solid blue line segments), and creates a mapping that preserves the strict monotonicity of the input data (purple dotted line). To obtain this new mapping, we interpolate between the midpoints (purple stars) of each piecewise constant segment. In practice, we find that both methods of calibration produce equally well-calibrated classifiers; that is, after either calibration method, the data points in our simulated dataset that have a calibrated posterior sweep probability of $k\%$ are made up of approximately $k\%$ sweep simulations and $(100 - k)\%$ neutral simulations (see Supplementary Figure 1d-e, Supplementary Figure 2d-e). However, smoothed isotonic regression has the advantage of preserving strict monotonicity of posterior probabilities.

Supplementary Figure 4

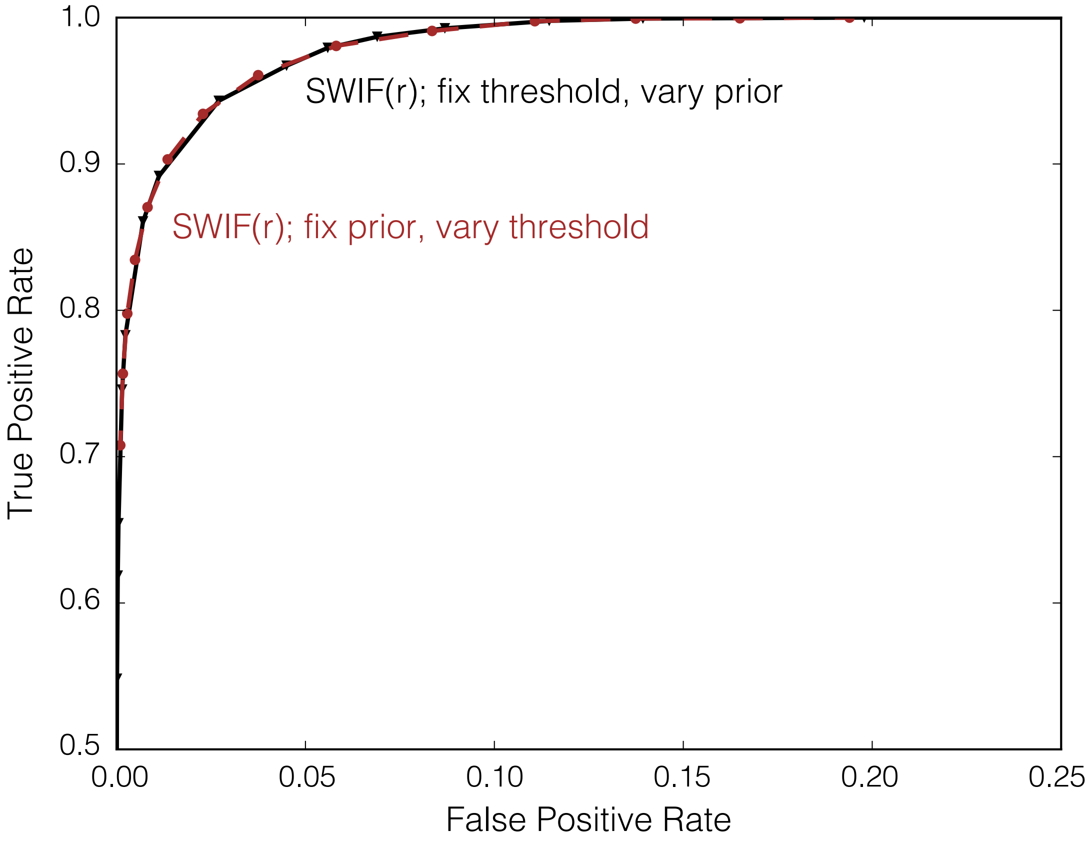

ROC curves for SWIF(r), varying the prior and varying thresholds. We plot ROC curves for SWIF(r) by fixing the threshold for classifying a site as adaptive at 50% posterior probability and varying the prior in Equation 3 (black curve with points denoted as triangles). However, an equally valid method would be to fix the prior and vary the posterior probability cutoff for determining which sites are classified as neutral and which are classified as adaptive (brown curve with circular points, prior fixed at 10^{-5}). We show below that both methods result in equivalent ROC curves.

Supplementary Figure 5

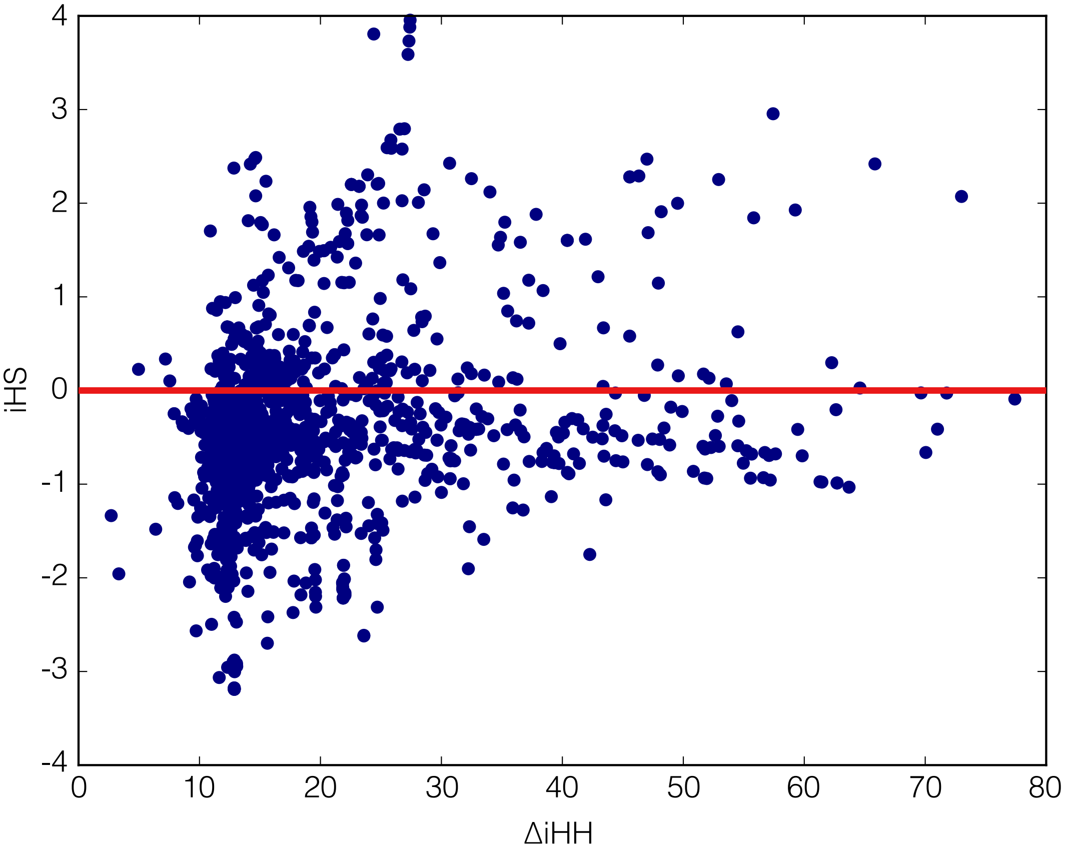

ΔiHH produces many false positives in 1000 Genomes data. Points represent ΔiHH and iHS values for sites classified as adaptive mutations (sweep probability $\geq 50\%$) in the 1000 Genomes data (here, ΔiHH included as a component statistic in SWIF(r)). The red line denotes iHS $= 0$, to emphasize the large number of these sites for which iHS is positive. Since positive values of iHS provide evidence *against* positive selection for the derived allele, these sites are likely false positives driven by ΔiHH.

Supplementary Figure 6

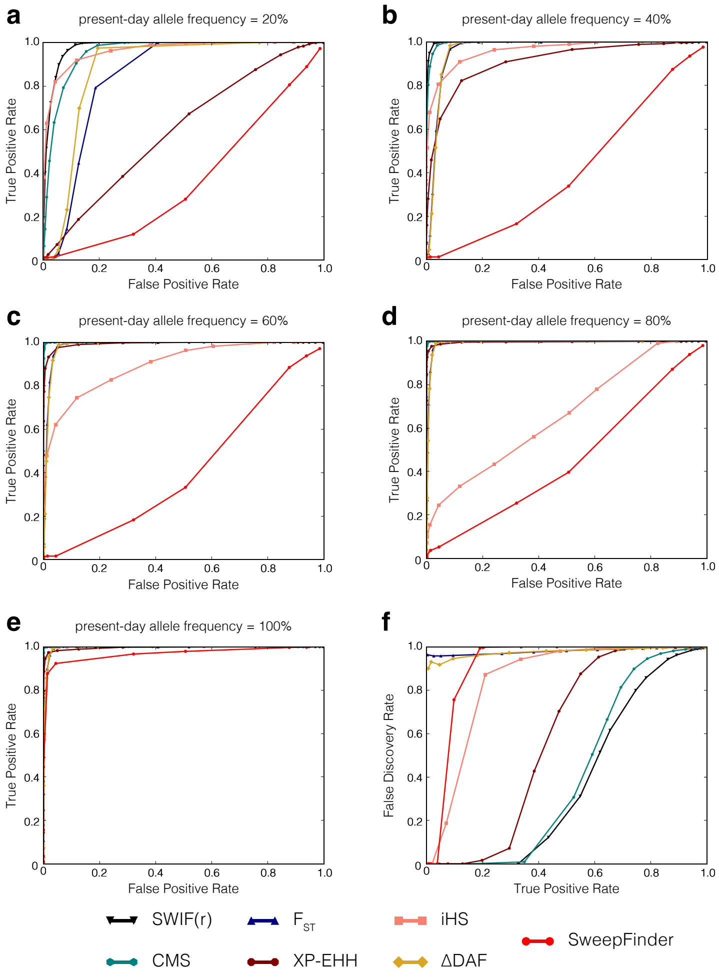

SWIF(r) outperforms sweep-detection method SweepFinder. SweepFinder[3] is a composite-likelihood method for detecting selective sweeps that is designed to be robust to recombination rate and demography. SweepFinder uses a theoretical model of changes in the allele frequency spectrum after a sweep that is dependent on the distance at each site to the site of the selective sweep, and is designed to detect completed sweeps (present-day beneficial allele frequency of 100% in the population of interest). **a-e)** As expected given SweepFinder's underlying model, the ROC curves for detecting incomplete sweeps (present-day beneficial allele frequencies from 20% to 80%) show very poor performance of SweepFinder relative to other methods. SweepFinder performs better for completed/nearly-completed sweeps relative to incomplete sweeps, but still lags behind other methods including SWIF(r). We note that iHS is missing from the plot of completed sweeps (panel e), because iHS cannot be calculated at such sites[4]. **f)** Power-FDR curves aggregated over all sweep parameters, assuming a training set composed of 99.95% neutral variants and 0.05% adaptive variants.

Supplementary Figure 7

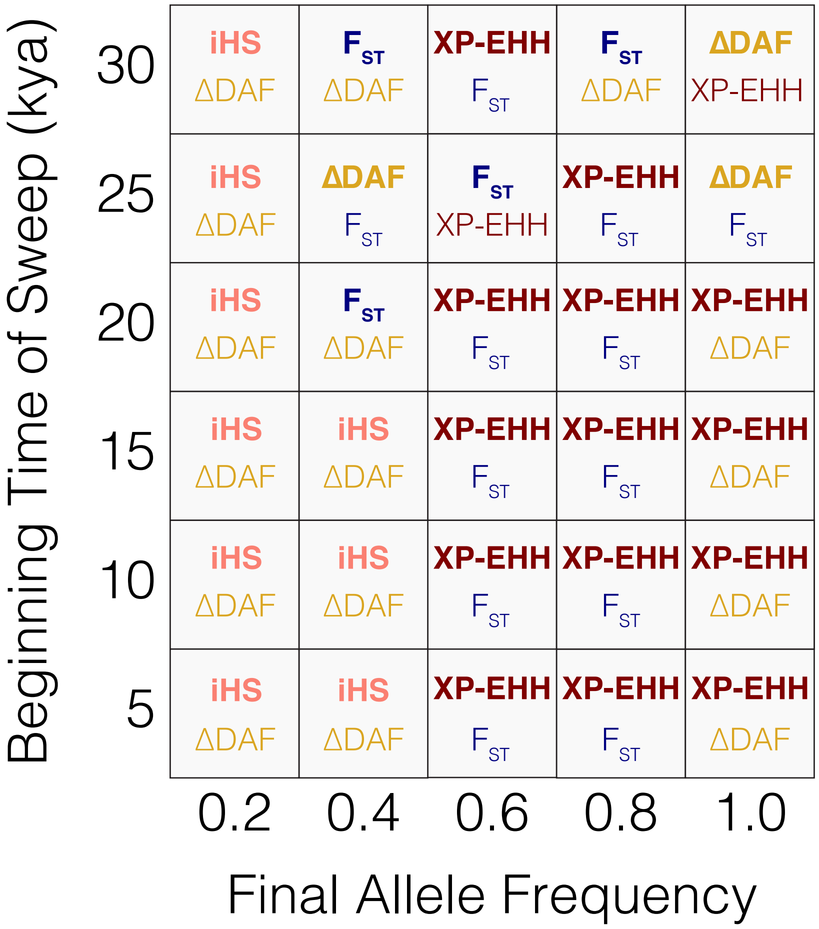

Component selection statistic rankings per sweep model parameters used in this study. Each box contains the top two performing component statistics (listed in rank order) for each pair of sweep start time and present-day frequency of the adaptive allele in the population of interest. Performance is evaluated as the area under the ROC curve. Colors correspond to those in Figure 1.

Supplementary Figure 8

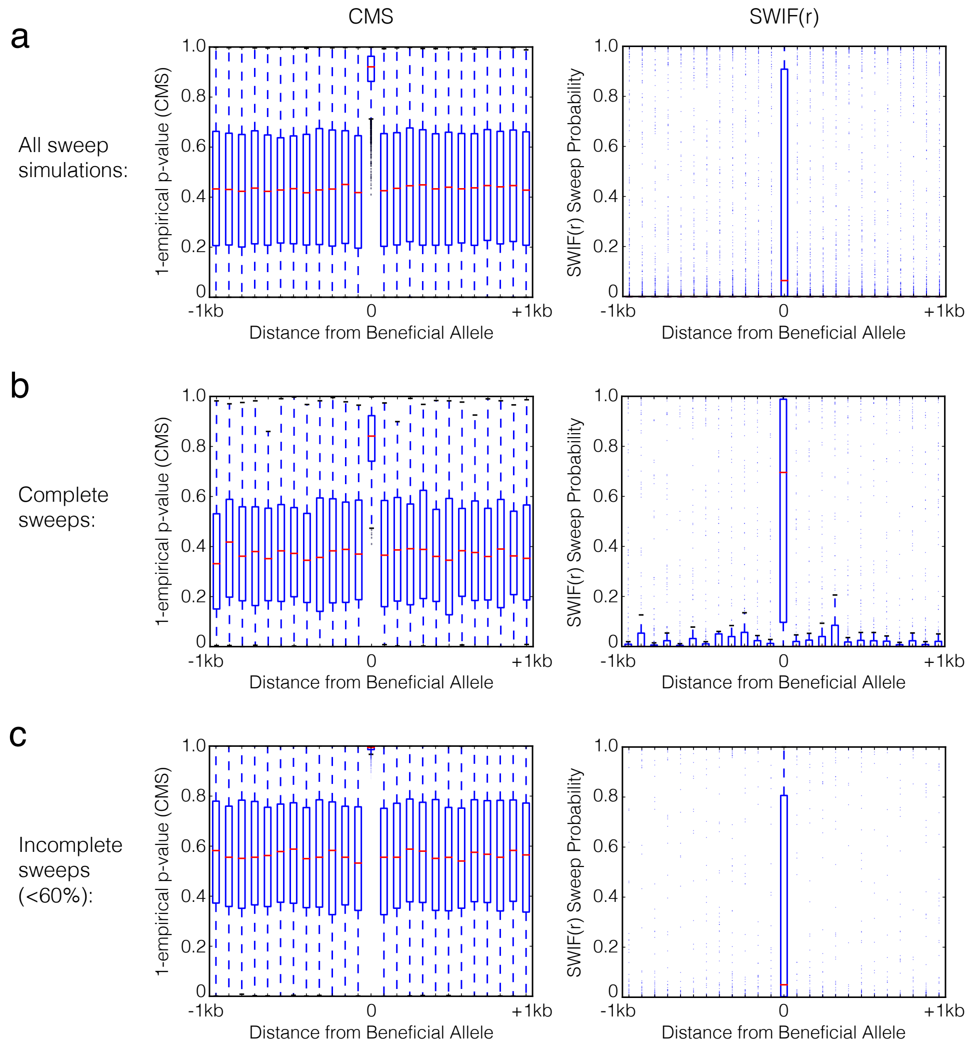

SWIF(r) outperforms CMS in localization of adaptive mutations in simulation. Boxplots show empirical p-values calculated with respect to all neutral simulations for CMS (left) and sweep probabilities returned by SWIF(r) (right). Center boxplots contain only adaptive sites, and all other boxplots evenly bin genomic coordinates 1kb up- and downstream of the adaptive SNP. Results are shown for **a)** all sweep parameters, **b)** complete sweeps (beneficial allele has reached a frequency of 100% in the population of interest), and **c)** incomplete sweeps where the beneficial allele has reached a maximum frequency of 60%. In each case, the distribution of CMS scores at the beneficial allele differs from the distributions at neighboring sites, but with significant overlap, making it difficult to confidently and quantitatively localize the adaptive site. In contrast, the distributions of SWIF(r) probabilities at neighboring loci are extremely low with little variance, with only outliers overlapping the range of probabilities seen at adaptive sites.

Supplementary Figure 9

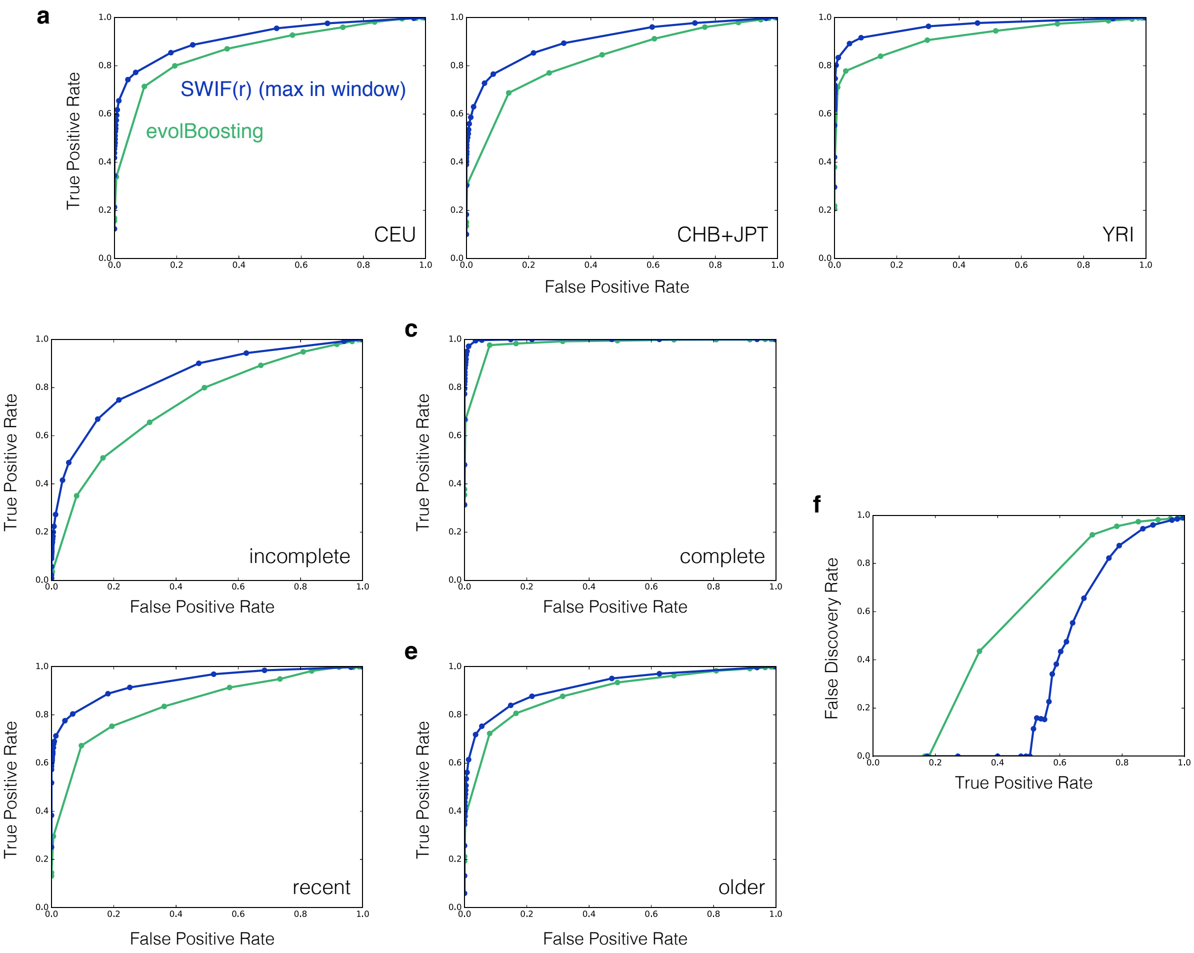

SWIF(r) outperforms evolBoosting[5]. Following Lin *et al.* [5], we implemented their software evolBoosting (`http://www.picb.ac.cn/evolgen/softwares/`) and applied it to simulated data in 40kb windows. For comparison, we calculated "window-based" SWIF(r) scores by taking the maximum site-based SWIF(r) probability in each 40kb window. We tested and trained evolBoosting on the middle 40kb of the 1Mb neutral simulations and sweep simulations described in Methods, for three populations modeled after CEU (Europe), CHB and JPT (East Asia), and YRI (West Africa). For a given threshold α, the false positive rate is defined as the fraction of neutral windows with a score above α, and the true positive rate is the fraction of windows containing a sweep with a score above α. The ROC curves below are obtained by varying α. **a)** Aggregated over all sweep parameters (beginning time of sweep between 5 and 30kya, present-day allele frequency between 20 and 100%), window-based SWIF(r) outperforms evolBoosting in all three populations. Results in remaining panels are averaged over populations. **b)** ROC curves for incomplete sweeps (present-day beneficial allele frequencies of 20-40%). **c)** ROC curves for complete and near-complete

sweeps (present-day beneficial allele frequencies of 80-100% in the population of interest). **d)** ROC curves for sweeps beginning 5-10kya. **e)** ROC curves for sweeps beginning 25-30kya. The performance of the two methods is closest for this parameter set representing older sweeps, likely reflecting the decreased power of haplotype-based statistics, which SWIF(r) relies on, relative to SFS-based statistics, which evolBoosting relies on. **f)** Power-FDR curves aggregated over all sweep parameters, assuming 1% of windows contain a sweep. We note that window-based methods can appear to have lower false discovery rates than site-based statistics, since there are many fewer windows than variants, and thus fewer opportunities for false positives to arise. However, this comes at the cost of a longer list of putative SNP targets, since each classified window contains a large number of individual variants.

Supplementary Figure 10

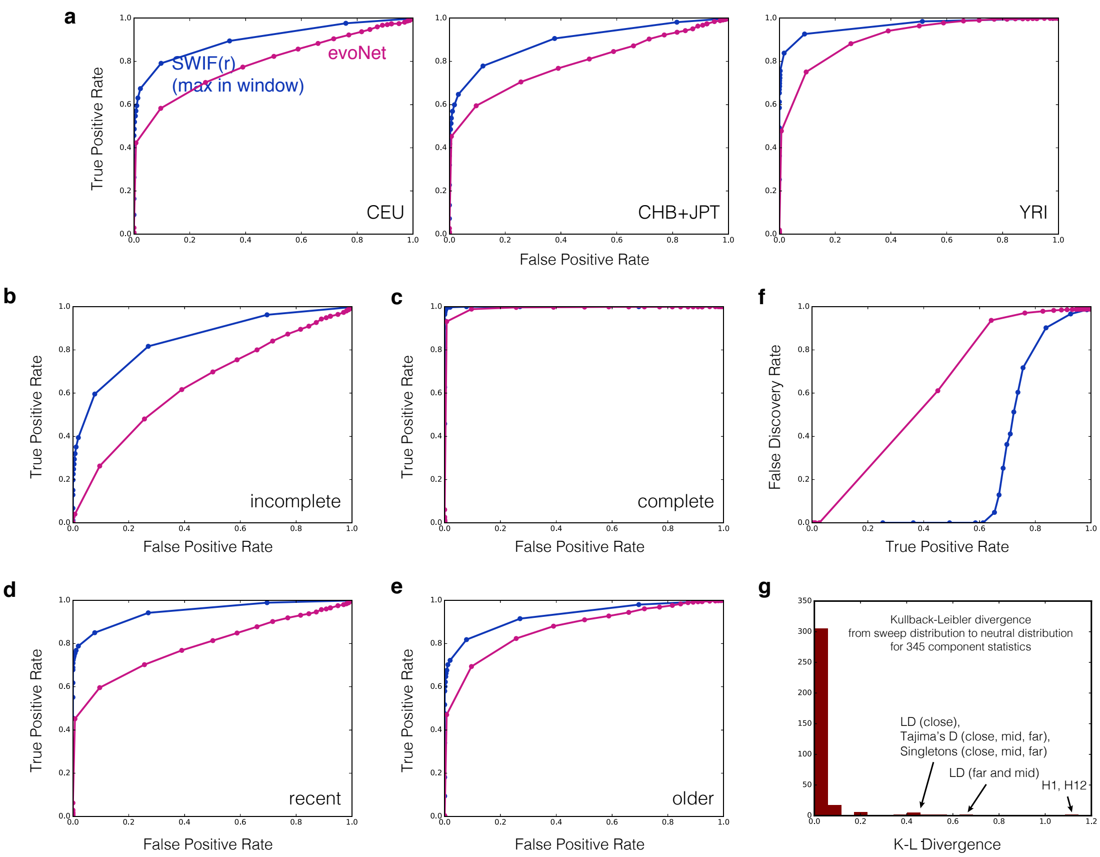

Performance comparison of SWIF(r) against evoNet[6]. evoNet[6] is a deep learning framework for simultaneous demographic and selection inference that uses 345 component statistics. Following Sheehan *et al.*[6], we implemented evoNet in 100kb windows, using the central 100kb of the 1Mb simulations described in Methods. For comparison, we calculated "window-based" SWIF(r) scores by taking the maximum site-based SWIF(r) probability in the same 100kb windows. For a given threshold α, the false positive rate is defined as the fraction of neutral windows with a score above α, and the true positive rate is the fraction of windows containing a sweep with a score above α. The ROC curves below are obtained by varying α. We note that these analyses likely downplay the strengths of SWIF(r) as a site-based classifier, and also do not reflect the power that evoNet has for jointly inferring demography and selection, which it was designed to do. **a)** Aggregated over all sweep parameters (beginning time of sweep between 5 and 30kya, present-day allele frequency between 20 and 100%), window-based SWIF(r) outperforms evoNet in all three populations. Results in remaining panels are averaged over populations. **b)** ROC curves for incomplete sweeps (present-day beneficial allele frequencies of 20-40%). **c)** ROC curves for complete sweeps (present-day beneficial allele frequencies of 80-100% in the population of interest). **d)** ROC curves for sweeps beginning

5-10kya. **e)** ROC curves for sweeps beginning 25-30kya. **f)** Power-FDR curves aggregated over all sweep parameters, assuming 1% of windows contain sweeps. We note that window-based methods can appear to have lower false discovery rates than site-based statistics, since there are many fewer windows than variants, and thus fewer opportunities for false positives to arise. However, this comes at the cost of a longer list of putative SNP targets, since each classified window contains a large number of individual variants. **f)** Given that evoNet uses 345 statistics while SWIF(r) uses only four, the superior performance of SWIF(r) is surprising at a glance; we believe that this is likely due to the fact that the majority of the 345 statistics are not informative for selection (but are likely informative for demographic history, which evoNet also infers). To demonstrate this, for each statistic, we have computed the Kullback-Leibler (K-L) divergence from the sweep distribution to the neutral distribution of the statistic calculated in simulations. A K-L divergence near zero represents near-total overlap of the two distributions, which indicates that the statistic is not informative for separating neutral and sweep windows. We find that for the vast majority of the 345 component statistics, the K-L divergence is near zero; a few exceptions are the H statistics developed by Garud *et al.*[7] to detect hard and soft sweeps, a few measures of linkage disequilibrium, number of singletons, and Tajima's D[8].

Supplementary Figure 11

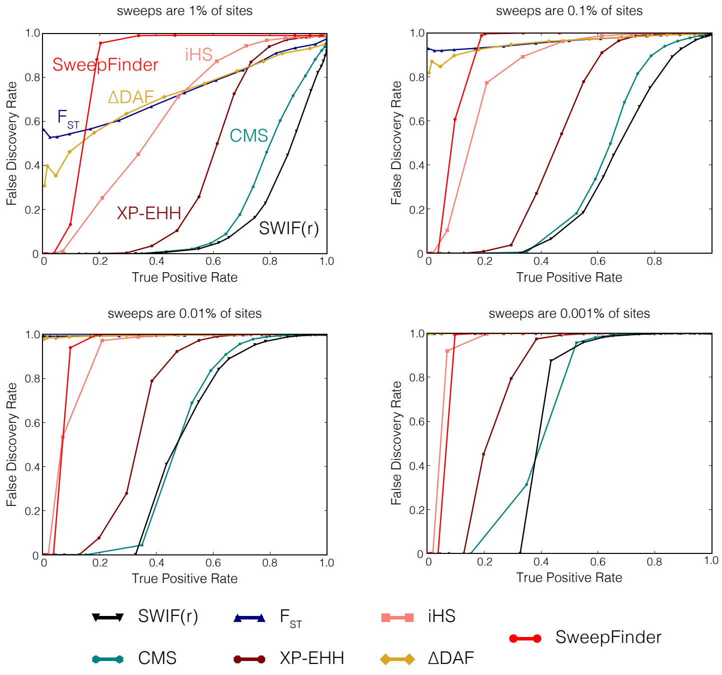

Power versus false discovery rate (FDR) for various training set compositions. While ROC curves are informative for illustrating the performance of different sweep detection methods, it is important to note that the genome has far more neutral variants than adaptive mutations. Therefore, more relevant performance comparisons can be made by illustrating the predicted false discovery rate (FDR) for a given true positive rate using Power-FDR curves. Using the same sets of simulations for testing and training that we use to generate the ROC curves in Figure 1, we calculated the tradeoff between true positive rate (power; the fraction of simulated sweep sites correctly classified as a sweep) and the false discovery rate (the fraction of sites classified as a sweep that are actually neutral). We note that the shape of these curves depends heavily on the makeup of the training set, since the false discovery rate rises as the proportion of adaptive variants in the training set decreases. Below we provide four sets of curves for training sets that are 1%, 0.1%, 0.01%, and 0.001% sweep sites respectively, with the remainder of the training set made up of neutral simulated sites. SWIF(r) performs well relative to CMS and the component statistics across training set compositions, in particular achieving a false discovery rate near zero for a moderate true positive rate of ∼30%. We note, however, that depending on the training set makeup, the false discovery rates for all methods can be quite high; we believe that this illustrates the difficulty of detecting selection reliably at a genome scale. We note that these curves were generated before calibration, but that calibrating SWIF(r) would not alter these curves, since the Power-FDR curves themselves are transformation invariant[9].

Supplementary Figure 12

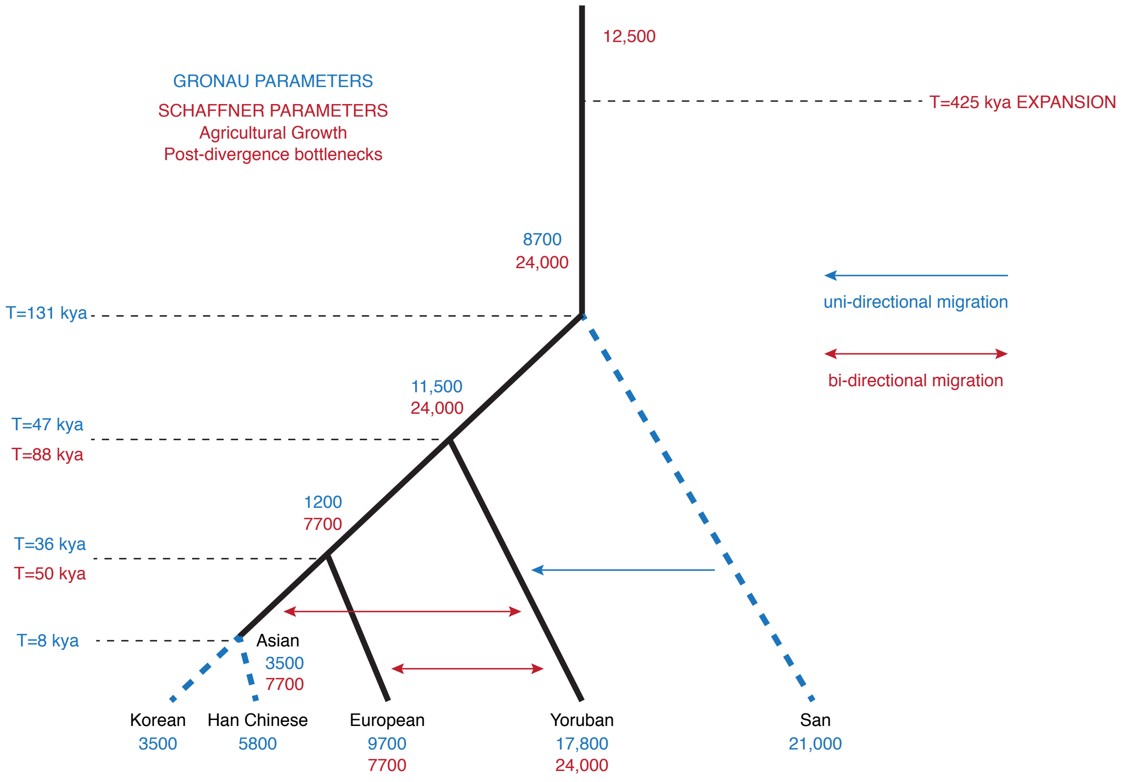

Illustration of differences between Gronau[10] and Schaffner[11] demographic models. Black lines indicate lineages inferred by both models, and blue dotted lines are lineages only inferred by Gronau *et al.*[10]. The models differ in many evolutionary parameter estimates: the Schaffner model includes an ancient population expansion and post-divergence bottlenecks, features not included in the Gronau model. Divergence times differ almost two-fold in some cases between the two models, with the Yoruban/Eurasian split at 47kya in the Gronau model and 88kya in the Shcaffner model. Furthermore, the Schaffner model allows migration between East Asian, European, and West African populations, while the Gronau model does not. Effective population sizes also differ dramatically, in some cases over six-fold.

Supplementary Figure 13

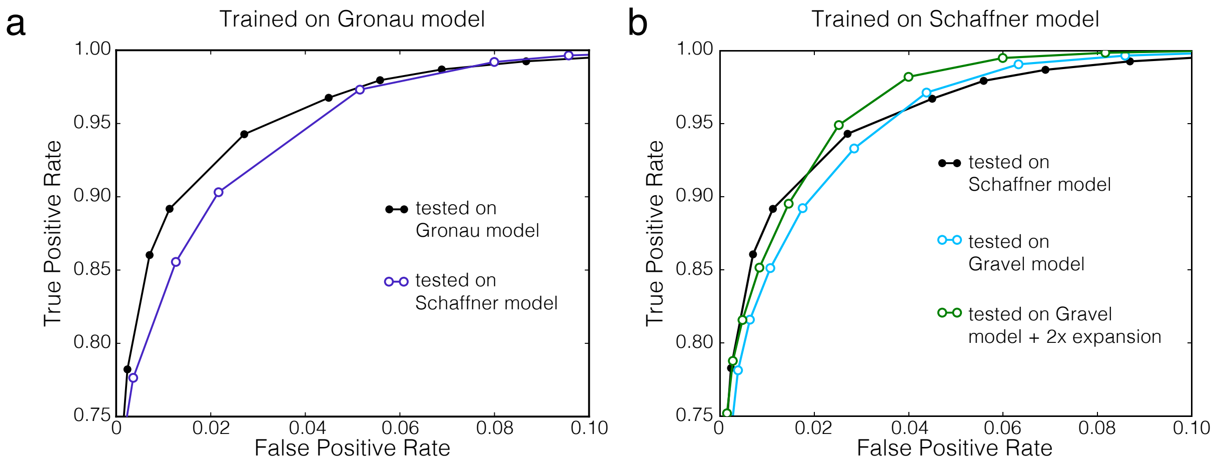

SWIF(r) is robust to demographic misspecification. We conducted three tests of robustness to demographic misspecification, using ROC curves to compare the performance of SWIF(r) when demographic models used for training and testing are the same (closed circles) and when they differ (open circles). **a)** To test the robustness of SWIF(r) to misspecification of divergence times, post-divergence bottlenecks, migration events, and ascertainment bias, we trained SWIF(r) on simulations from the Gronau model[10] (including ascertainment modeling), and tested the resulting classifier on two datasets: 1) simulations from the same demographic model (black curve), and 2) simulations from the Schaffner model with no growth (dark blue curve; see Supplementary Figure 12). Misspecification of these demographic parameters, and of ascertainment bias, does not have a large effect on the overall performance of SWIF(r). **b)** To test the robustness of SWIF(r) to population expansion, we first trained SWIF(r) on simulations from the Schaffner[11] demographic model with no growth, then tested the resulting classifier on three datasets: 1) simulations from the same demographic model (black ROC curve), 2) simulations from the demographic model from Gravel *et al.*[12], which incorporates recent exponential population expansion (light blue curve), and 3) simulations from an altered version of the Gravel demographic model in which we double the rates of recent exponential expansion (from 0.38% to 0.76% for Europe, and from 0.48% to 0.96% for East Asia), resulting in 15-fold and 30-fold increases in present-day population sizes for Europe and East Asia respectively (green curve). We show here that SWIF(r) is robust to dramatic misspecification of population sizes and population size changes. Note that false positive rate ranges from 0% to 10% in both panels.

Supplementary Figure 14

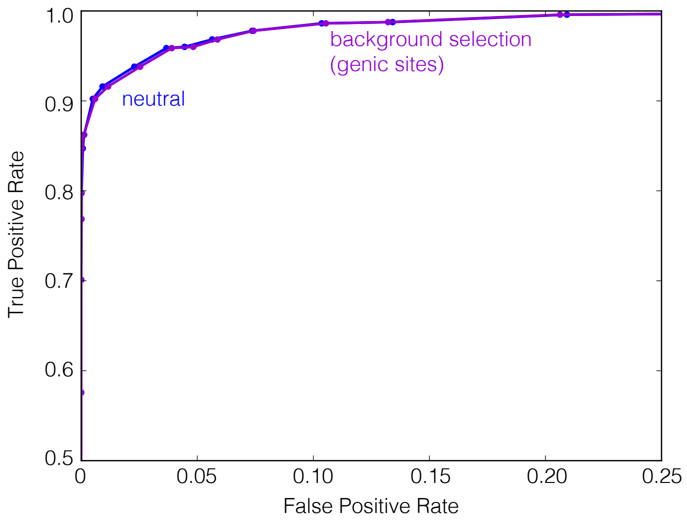

SWIF(r) is robust to background selection in simulation. To test the ability of SWIF(r) to differentiate between selective sweeps and background selection, we generated simulated data for neutral, sweep, and background selection regions using forward simulator `slim`[13]. We followed Messer *et al.*[14] for simulating background selection: briefly, we simulated genes with 8 exons of 150bp each, separated by introns of 1.5kb each, and surrounded by 550bp and 250bp 5′ and 3′ UTRs, respectively. We assumed that 75% of sites in exons and UTRs were functional (subject to purifying selection), that mutations were codominant, and that fitness effects at different sites were additive. 40% of functional sites were modeled as strongly deleterious ($s = -0.1$), and the rest were weakly deleterious with selection strengths ranging between -0.01 and -0.0001 (see Messer *et al.*[14]). For sweep simulations, selection coefficients were drawn from an exponential distribution with mean 0.03, and sweeps began 10,000 years ago. For all simulations, we modeled two populations with $N_e = 5000$, with a population split 40,000 years ago, and we used a mutation rate of 2.5×10^{-8} and recombination rate of 10^{-8} (also following Messer *et al.*[14]). We trained SWIF(r) using neutral and sweep simulations, and then tested the performance of the resulting classifier in distinguishing neutral variants from sweep variants (blue), and distinguishing genic (UTR, exonic, and intronic) variants from sweep variants (purple). True positive rate is the fraction of simulated sweep variants that are correctly classified, and false positive rate is the fraction of neutral (blue) or genic (purple) variants that are incorrectly classified as adaptive. These results indicate that SWIF(r) is completely robust to background selection, which is a function of the component statistics it uses; Enard *et al.*[15] have shown that iHS and XP-EHH are robust to background selection, and since deleterious alleles are unlikely to rise to high frequency (indeed, are less likely to do so than neutral variants), we would expect that F_{ST} and ΔDAF would also be robust.

Supplementary Figure 15

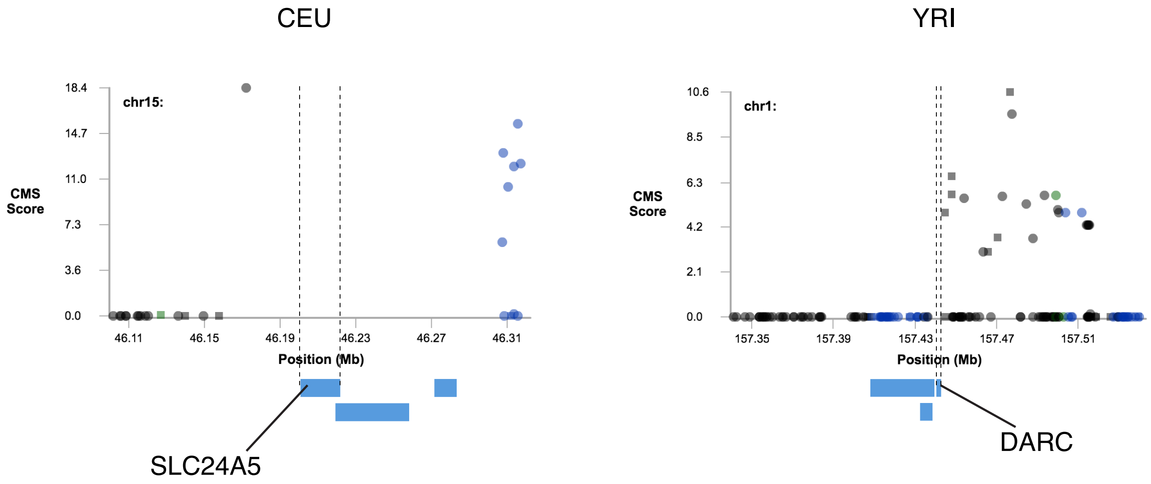

CMS scores on known targets of selective sweeps. Plots made with CMSviewer (`https://pubs.broadinstitute.org/mpg/cmsviewer/` use date 04/26/2016) show CMS scores[16] around *SLC24A5* in CEU and *DARC* in YRI. In each case, SNPs within the gene (region shown between dotted lines) are not assigned CMS scores, possibly due to undefined component statistics. Indeed, in our scan for selection using SWIF(r), iHS and ΔiHH are undefined at the causal mutation in *DARC* and throughout *SLC24A5*. We were unable to obtain plots for *OCA2* and *EDAR* from CMSviewer on subsequent use dates.

Supplementary Figure 16

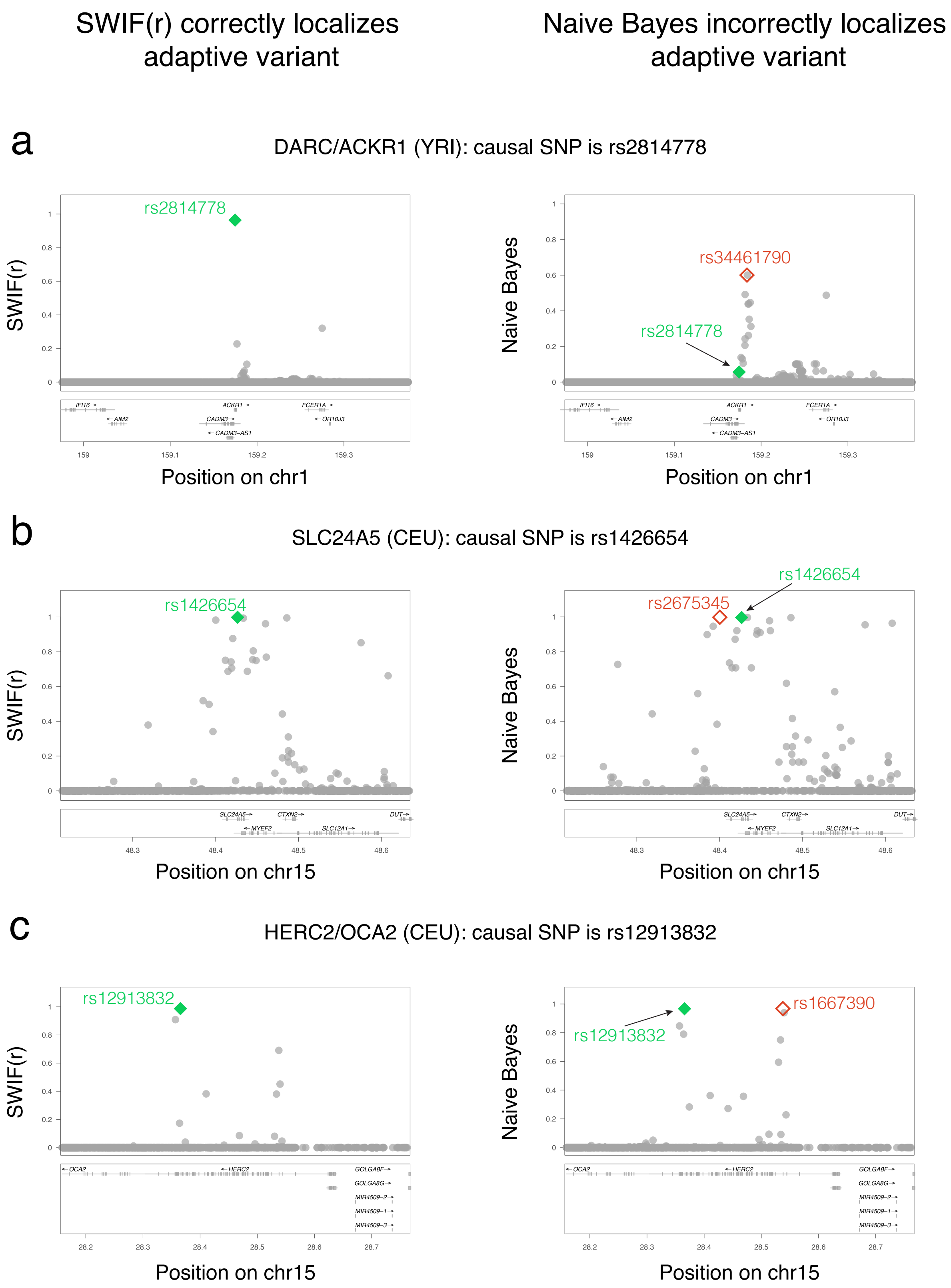

Joint distributions are necessary for localization of previously validated adaptive SNPs. We note that if we do not learn joint distributions and only univariate distributions, then the AODE reduces down to a Naive Bayes classifier, which is very similar to CMS[17] except that the Naive Bayes classifier returns a probability. Here we compare SWIF(r) to a Naive Bayes classifier in order to illustrate what is gained by learning joint distributions. Posterior probabilities based on SWIF(r) and a Naive Bayes classifier are shown for three genes with validated adaptive SNPs, **a)** *DARC*, **b)** *SLC24A5*, and **c)** *HERC2*. In each case, SWIF(r) assigns the highest sweep probability in the region to the adaptive SNP (in green filled diamond, left column), while the Naive Bayes classifier does not. This is most dramatic for *DARC*, where the Naive Bayes we implemented classifier assigns the causal SNP a very low probability (in green diamond, right column, denoted by arrow), and a different SNP (in red open diamond) is assigned the highest probability. For *SLC24A5* and *HERC2*, the Naive Bayes classifier assigns high probabilities to the causal SNPs, but cannot distinguish these from other similarly high-scoring SNPs. See Figure 2 caption for citations concerning functional validation of these adaptive SNPs.

Supplementary Figure 17

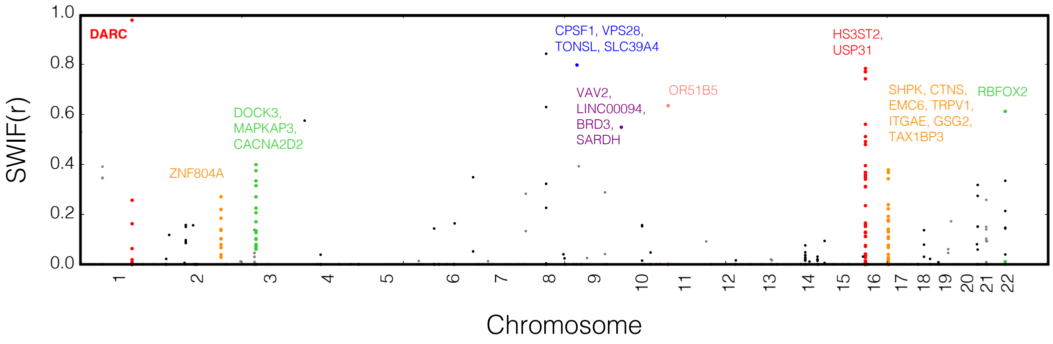

Full selection scan using SWIF(r) in the YRI population (1000 Genomes phase 1[18]). The value plotted for each position along the genome is the calibrated posterior sweep probability calculated by SWIF(r), with per-site prior $\pi = 10^{-5}$ (Supplementary Figure 1). Only SNPs with sweep probability greater than 1% are plotted. Genes containing a single SNP with sweep probability over 50%, or containing multiple SNPs with sweep probability over 10% are annotated. We note that a paralog of *HS3ST2* (*HS3ST3A1*) has been linked to malaria resistance[19].

Supplementary Figure 18

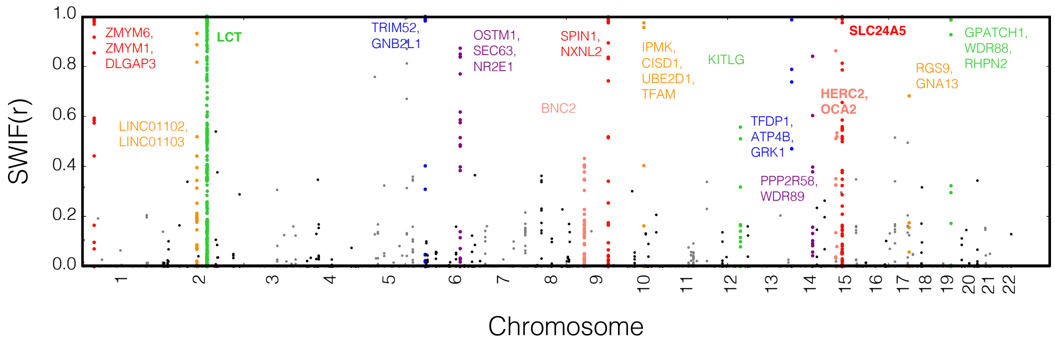

Full selection scan using SWIF(r) in the CEU population (1000 Genomes phase 1[18]). The value plotted for each position along the genome is the calibrated posterior sweep probability calculated by SWIF(r), with per-site prior $\pi = 10^{-5}$ (Supplementary Figure 1). Only SNPs with sweep probability greater than 1% are plotted. Genes containing a single SNP with sweep probability over 50%, or containing a cluster of SNPs with sweep probability over 10% are annotated. In addition to *SLC24A5* and *OCA2*, SWIF(r) also identifies other canonical targets of positive selection in Europeans, including *LCT*[20], *BNC2*[21], and *KITLG*[22].

Supplementary Figure 19

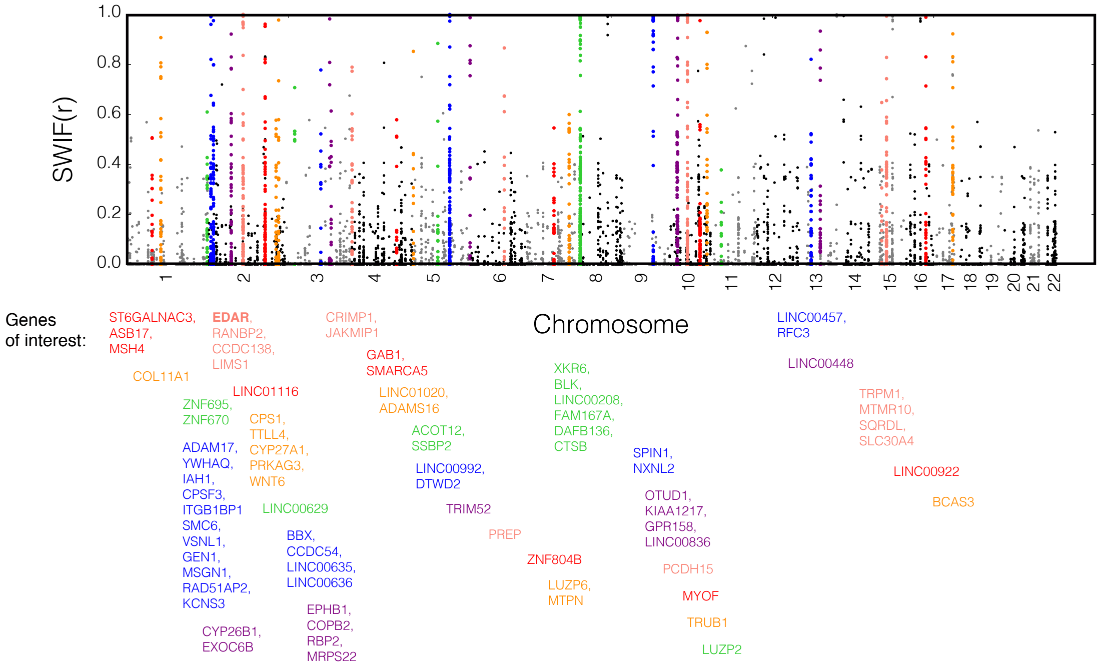

Full selection scan using SWIF(r) in the CHB and JPT populations (1000 Genomes phase 1[18]). The value plotted for each position along the genome is the calibrated posterior sweep probability calculated by SWIF(r), with per-site prior $\pi = 10^{-5}$ (Supplementary Figure 1). Only SNPs with sweep probability greater than 1% are plotted. Genes containing a single SNP with sweep probability over 50%, or containing a cluster of SNPs with sweep probability over 10% are annotated. We suspect that the abundance of selection signals in this population is partly a consequence of longer LD blocks in East Asian populations relative to European and West African populations, and partly a consequence of the fact that the simulation software `cosi`[11] precludes modeling demographic events during the duration of a sweep, and so very recent population expansions are not modeled in our simulations; Supplementary Figure 20 shows that the distributions of component statistics across populations differs more in observed 1000 Genomes data than in our demographic simulations. We also note that this abundance of signal in East Asian populations has been previously observed[23].

Supplementary Figure 20

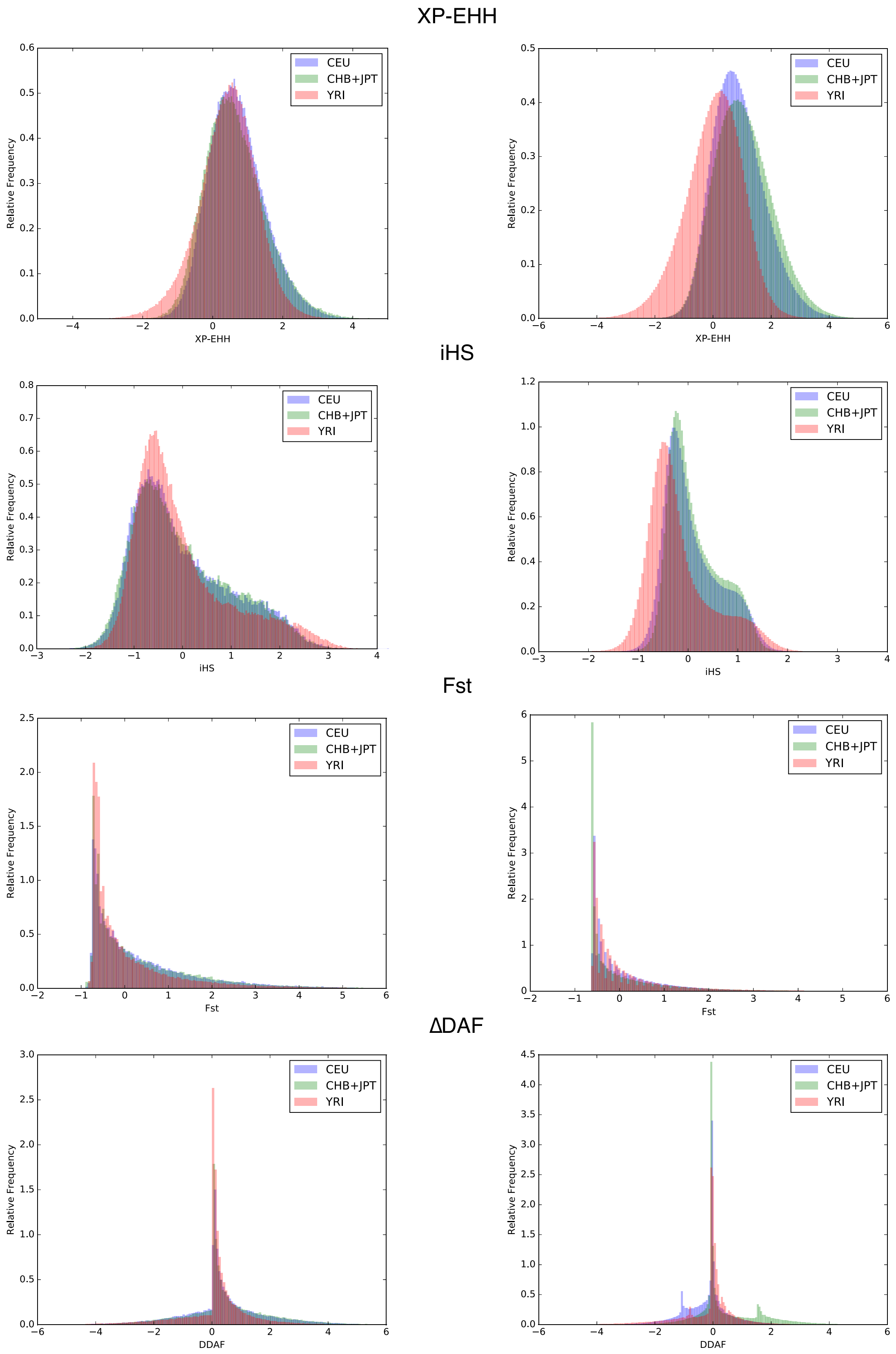

Component statistic distributions for 1000 Genomes data and simulations suggest reasons for discrepancy in number of SWIF(r) targets across populations. The left column shows histograms of component statistic values in simulated data using the demographic model from Schaffner *et al.*[11], and the right column has the corresponding histograms for the 1000 Genomes data. XP-EHH shows greater bias by population in real data than in simulated data, even after normalizing with population-specific mean and variance based on simulations, with more negative values in the African population than in the other two. iHS shows a similar pattern, despite having been designed to minimize this bias[4]. The distributions of ΔDAF in real data also deviate substantially from simulations; the East Asian population in particular has values that are skewed higher than in the other two populations, possibly as a result of increased genetic drift[24]. These differences may be a consequence of the fact that our simulations did not include very recent population expansions, because of limitations of the simulation software `cosi` regarding the overlap of selective sweeps and demographic events[11]. Most of these differences likely have the effect of leading to an increase in the number of predicted sweep sites in East Asia, and a decrease in West Africa, as we observe in our scan using the 1000 Genomes data, and as has been observed previously[23]. Note that x- and y-axis limits vary for each panel.

Supplementary Figure 21

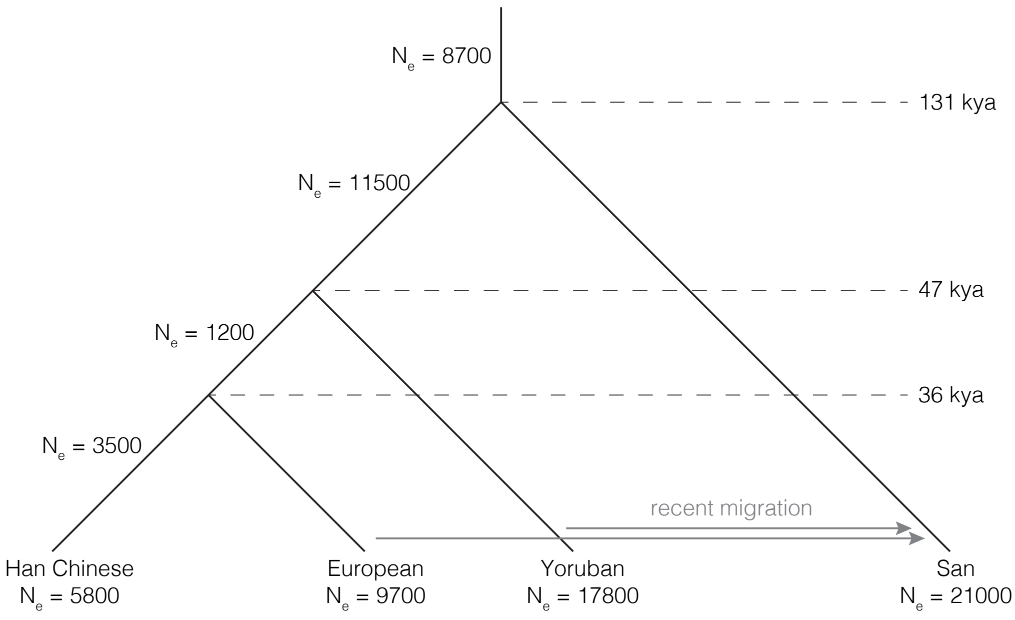

Demographic model used in analyses of the ‡Khomani San. Effective population sizes, coalescent times, and migration rate and times used for simulating haplotype data in analyses including the ‡Khomani San, adapted from Gronau *et al.*[10]. We adapted migration rates from Uren *et al.*[25]: a one-generation pulse from Europe with rate 0.197/chromosome/generation 7 generations ago, and a one-generation pulse from Yoruba with rate 0.227/chromosome/generation 14 generations ago (both shown with arrows in this diagram).

Supplementary Figure 22

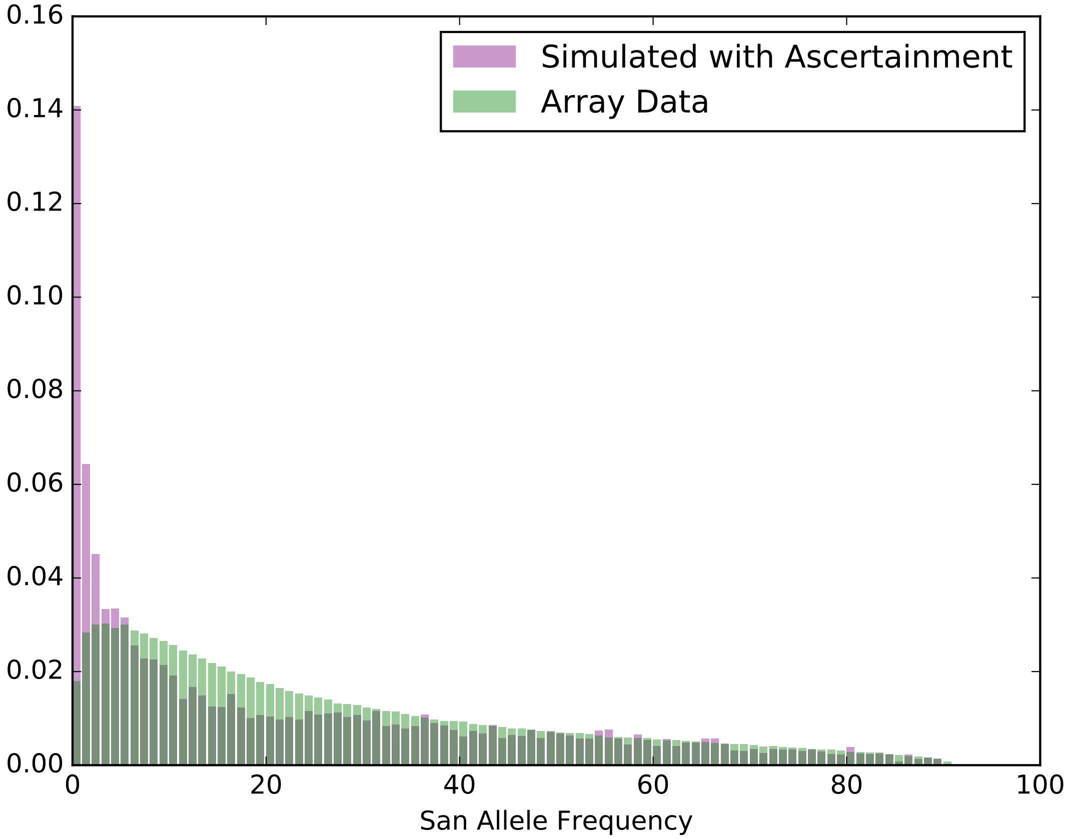

Site Frequency Spectrum of observed array data and simulated data with ascertainment for ‡Khomani San. After ascertainment on simulated haplotypes, we see fairly good agreement in these site frequency spectra, except for the abundance of very low-frequency derived alleles in simulations. We believe this to be because of standard quality filtering steps taken with array data that are not modeled in the ascertainment process. This difference should not affect the output of SWIF(r) for two reasons: first, we only consider derived mutations in the human lineage as potential sweep targets; and second, SNPs with very low derived allele frequencies are unlikely to register strong signatures of adaptive evolution. Supplementary Figure 23 shows that low-frequency SNPs do not affect the summary statistics used in our implementation of SWIF(r), including haplotype-based statistics like iHS.

Supplementary Figure 23

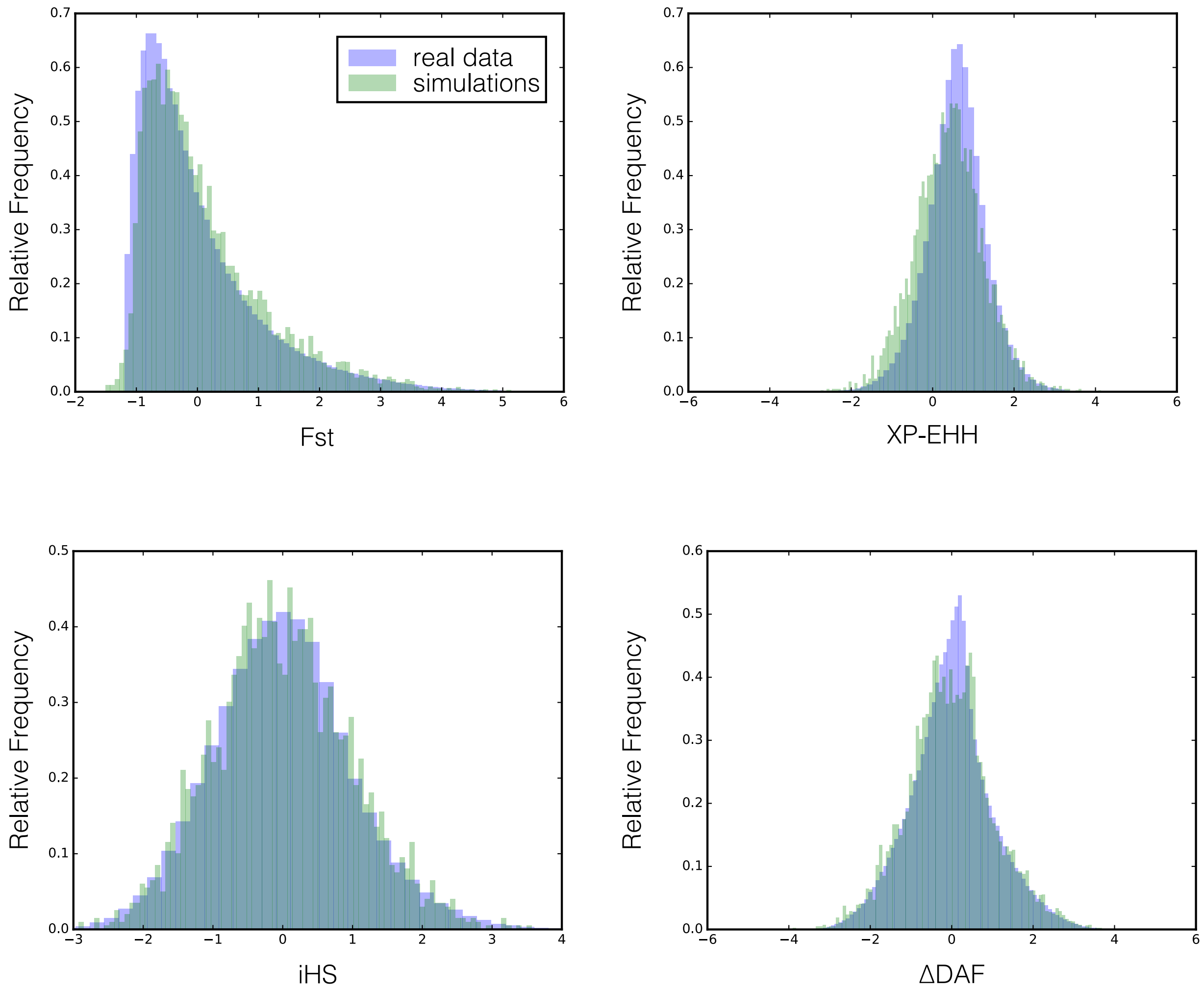

Component statistic distributions for ‡Khomani San simulations and observed data. Despite an excess of very low-frequency derived alleles in the simulated data (Supplementary Figure 22), the distributions of component statistics in simulations and in observed data are quite similar. We are therefore confident that this discrepancy does not inhibit the ability of SWIF(r) to detect selection in this population.

Supplementary Figure 24

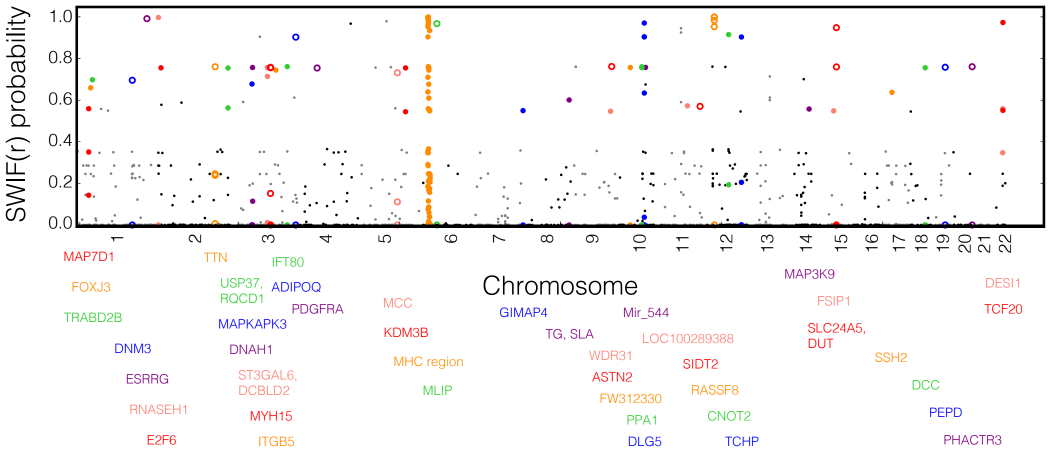

Full selection scan results from analyses of 45 ‡Khomani San samples. The value plotted for each position along the genome is the calibrated posterior sweep probability computed by SWIF(r), with per-site prior $\pi = 10^{-4}$ (Supplementary Figure 2) in order to detect signals of relatively old sweeps given the high long-term N_e of the ‡Khomani San. Only SNPs with posterior sweep probability greater than 1% are plotted. All genes containing at least one SNP with posterior sweep probability over 50% are labeled below the plot, and all variants within those genes are colored to match the gene label. A subset of these results are shown in Figure 3b; genes highlighted in that figure are denoted by open circles.

Supplementary Figure 25

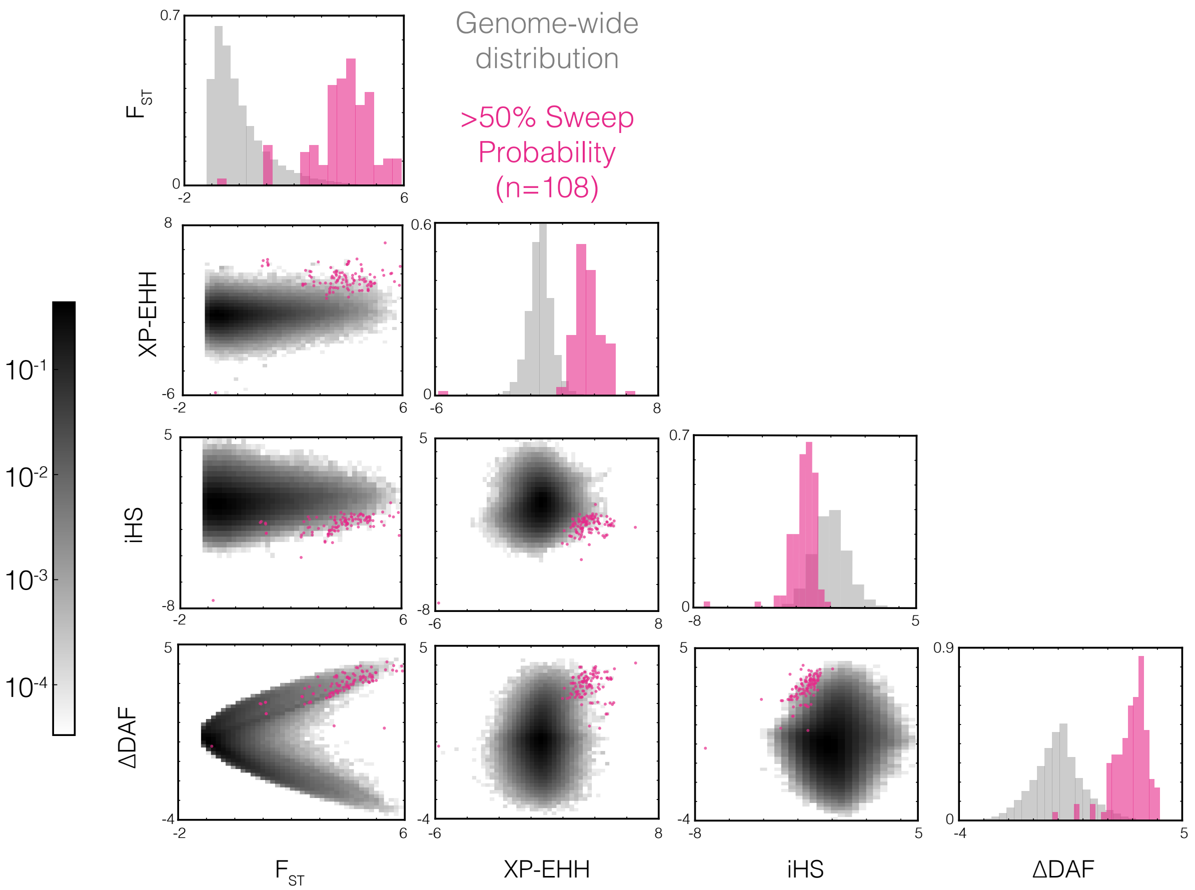

Genome-wide univariate and joint distributions of component statistics used to identify adaptive mutations in the ‡Khomani San, highlighting variants classified by SWIF(r) as adaptive. Distributions along the diagonal show the univariate empirical distributions for each component statistic used when applying SWIF(r) to genomic data from the ‡Khomani San, in gray for the whole genome, and in pink for sites classified as adaptive with a posterior sweep probability over 50%. Both histograms in each plot are normalized, with the genome-wide distributions made up of 628,032 sites for ΔDAF and F_{ST}, 624,834 for XP-EHH, and 538,928 for iHS. XP-EHH and iHS are calculated at fewer sites because the component statistics are undefined in cases where ΔDAF and F_{ST} are well-defined. Off-diagonal plots show the joint distributions of each pair of component statistics, again with the empirical genome-wide distribution in gray with a log scale shown in the colorbar, and the classified sweep sites in pink (SWIF(r) posterior probability ≥ 0.5). Any given joint distribution is plotted for only those sites at which both component statistics are defined.

Supplementary Figure 26

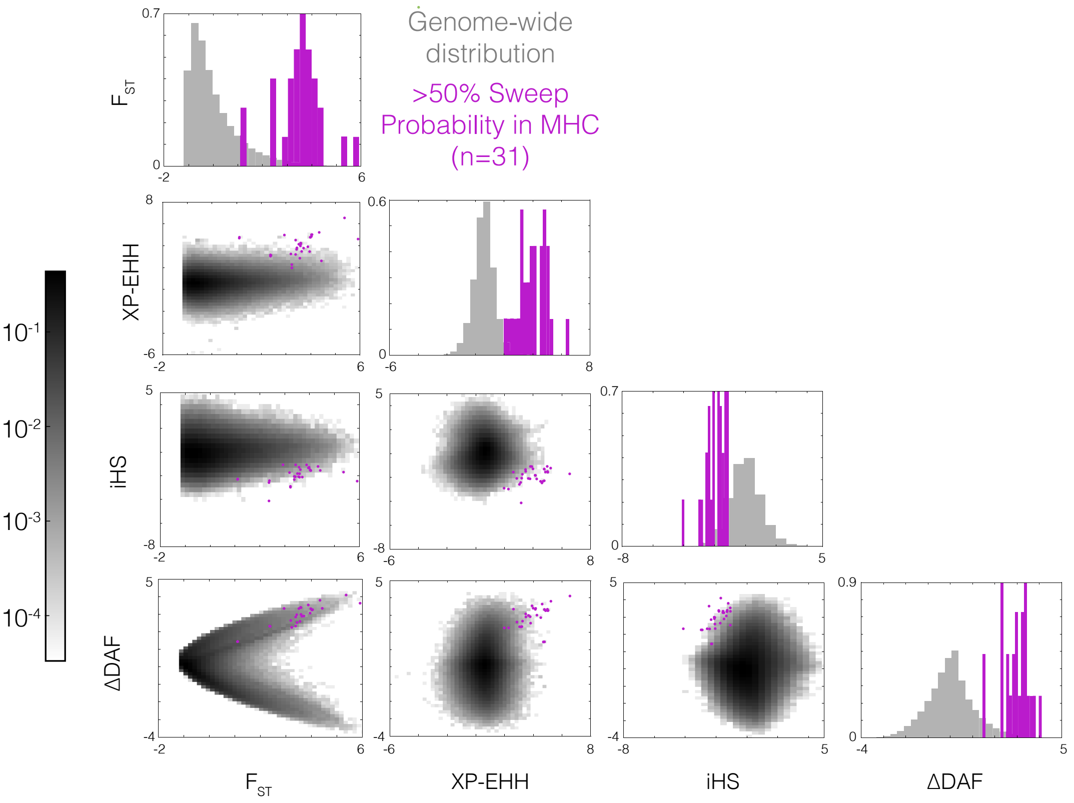

Univariate and joint distributions of component statistics used to identify adaptive mutations in the ‡Khomani San, highlighting SWIF(r) signals in MHC. Distributions along the diagonal show the univariate empirical distributions for each component statistic used when applying SWIF(r) to genomic data from the ‡Khomani San, in gray for the whole genome, and in green for sites within MHC classified as adaptive with a posterior sweep probability over 50%. Off-diagonal plots show the joint distributions of each pair of component statistics, again with the empirical genome-wide distribution in gray with a log scale shown in the color bar, and the classified MHC sites in green. Any given joint distribution is plotted for only those sites at which both component statistics are defined.

Supplementary Figure 27

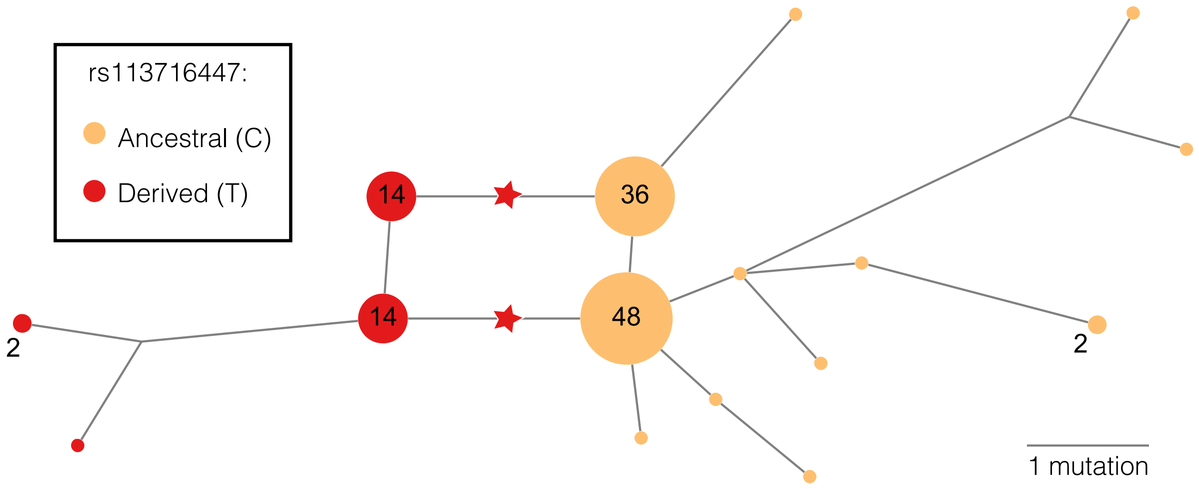

Haplotype network for *ADIPOQ* gene region in the ‡Khomani San dataset. The median-joining haplotype network[26] is built using the Network software package v4.6.1.1 (`fluxus-engineering.com`) from combined exome and SNP array data for the ‡Khomani San population. Haplotypes span 17 SNPs over ∼4kb from chr3:186571486-186575536, an interval containing *ADIPOQ*, pruned to avoid recombination hotspots. Nodes represent specific haplotypes and are annotated with the number of times each haplotype appears in the population. Node sizes are scaled relative to the number of individuals represented. Nodes without labels appear once in the population. Edge lengths are proportional to the number of mutations that distinguish the two nodes on either end, and starred edges are those that are defined by the missense mutation at rs113716447. Nodes are colored by whether the haplotype carries the ancestral or derived allele of that gene. Although the available data for this gene region is limited, this network is consistent with selection on the derived allele.

Supplementary Figure 28

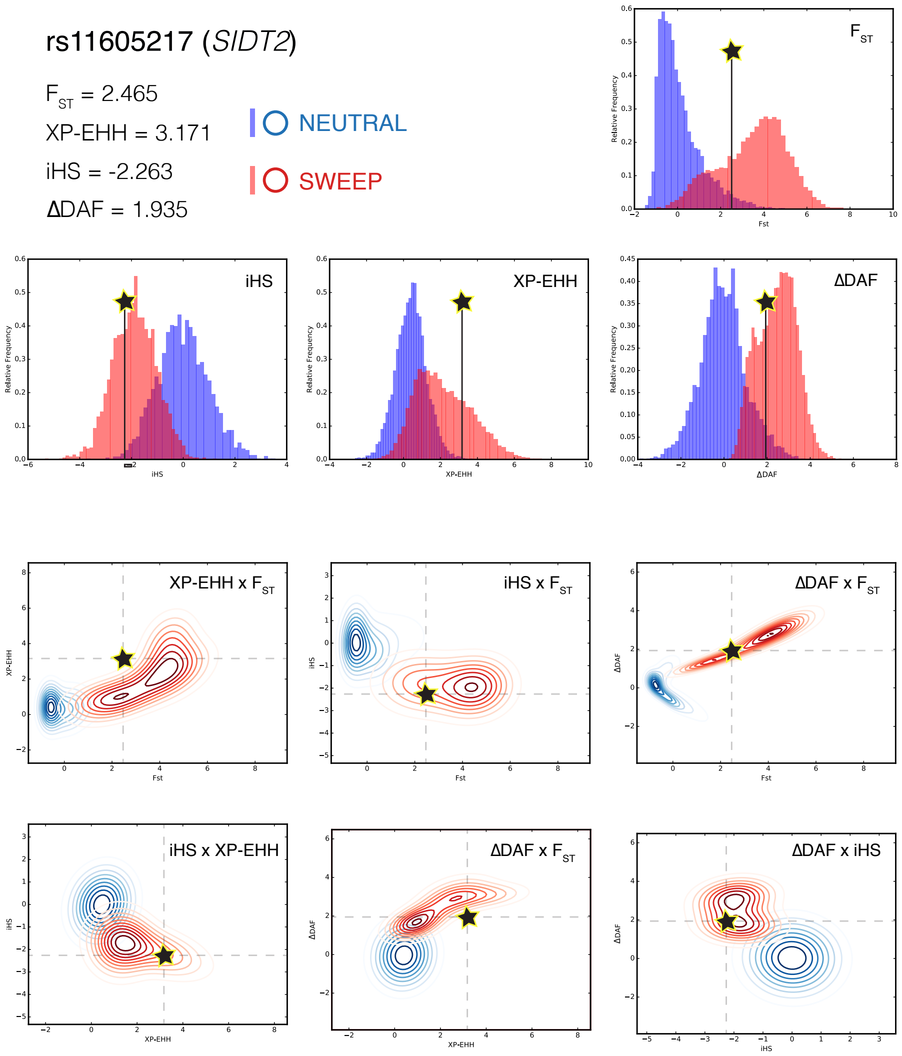

Knowledge of joint distributions allows for identification of *SIDT2* as a target of selection in the ‡Khomani San. The SNP identified by SWIF(r) as the site of an adaptive mutation in *SIDT2*, rs11605217 (posterior sweep probability 61%), had the following values for each summary statistic: F_{ST} = 2.456, XP-EHH = 3.171, iHS = -2.263, ΔDAF = 1.935. If we do not learn joint distributions and instead only learn univariate distributions (resulting in a Naive Bayes classifier), the posterior probability at this SNP is only 30%. While these statistics individually show moderate evidence for a sweep, their accumulated evidence in a Naive Bayes framework is not enough to overcome a low prior probability, and F_{ST} and ΔDAF in particular land well within normal limits for their respective neutral distributions (top). However, when we look at the pairwise joint distributions (bottom), in each case, the mutation in *SIDT2* lands within or nearby the sweep distribution, and relatively far from the neutral distribution.

Supplementary Figure 29

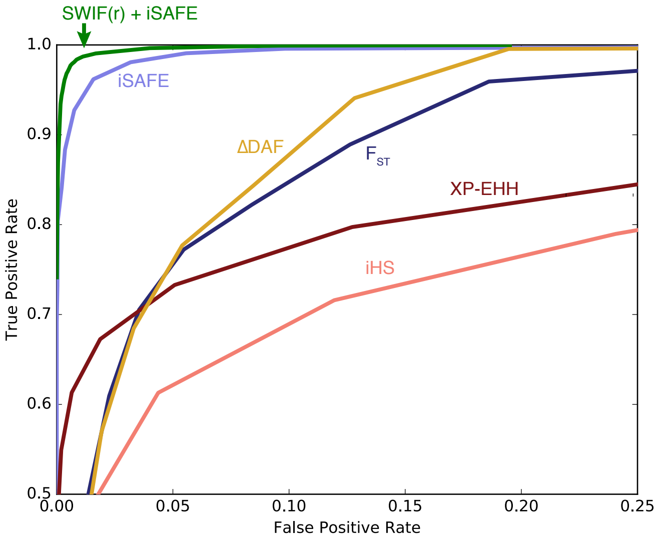

SWIF(r)'s framework can easily incorporate new statistics for increased power. A major feature of SWIF(r) is that it is completely generalizable to any set of component statistics, which means that SWIF(r) can take immediate advantage of more powerful statistics as they are designed. One such statistic, iSAFE[27] (preprint available at `https://www.biorxiv.org/content/early/2017/10/01/139055`), is an approach that ranks mutations within a genomic region that has been predetermined to contain a selective sweep. As shown below (light blue ROC curve), iSAFE is much more powerful than the component statistics used in this implementation of SWIF(r) at localizing beneficial mutations; by incorporating iSAFE as a component statistic, the overall performance of SWIF(r) improves and exceeds that of iSAFE, because SWIF(r) can leverage the joint distributions of iSAFE with other component statistics.

Supplementary Figure 30

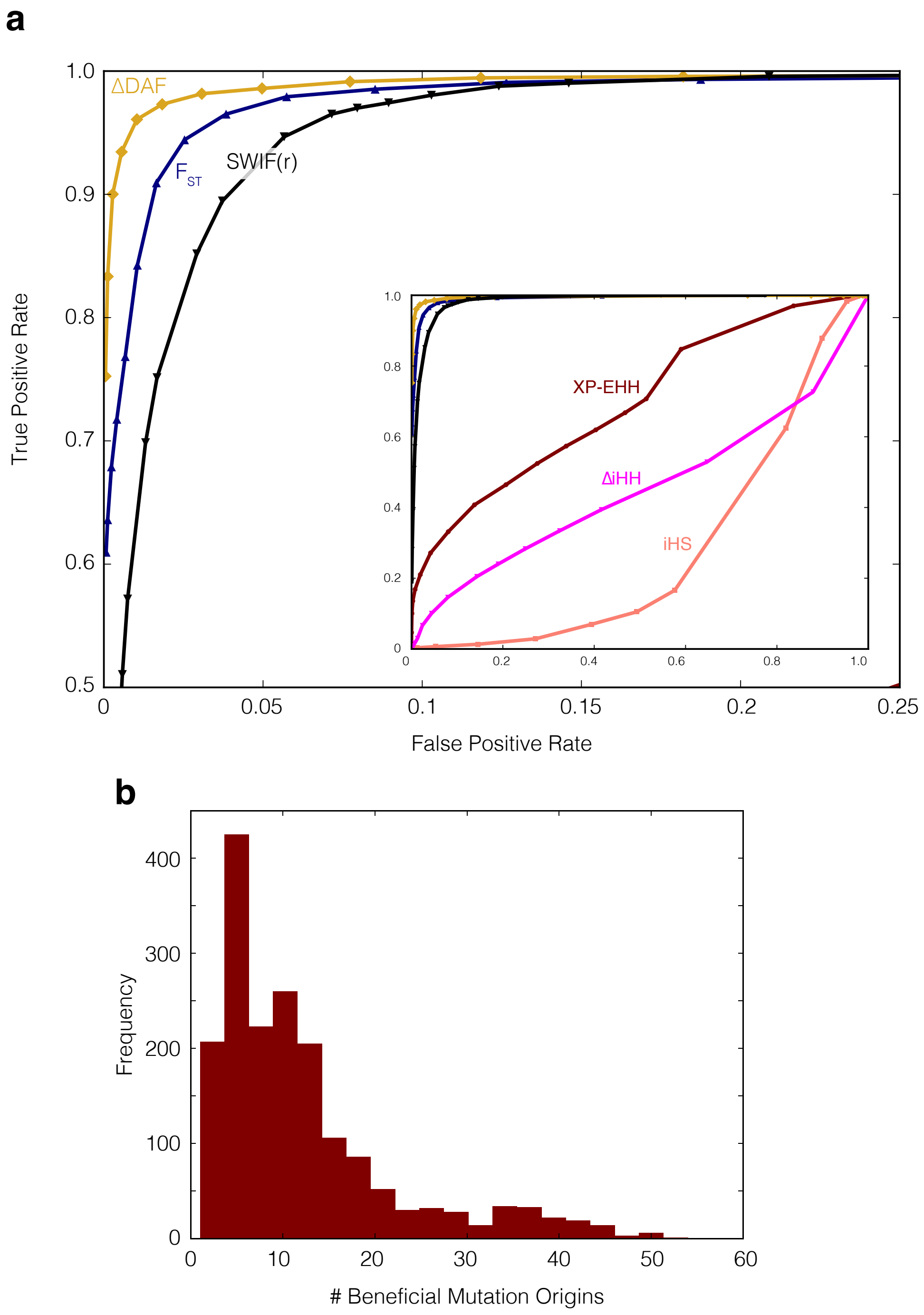

SWIF(r) also detects signatures of soft sweeps from standing variation, despite being trained on hard sweep simulations. Neutral and soft sweeps simulations were generated using `msms`[28] with parameters designed to match the Schaffner demographic model[11] (command line code: `java -jar msms/msms.jar -N 10000 -ms 360 1 -I 3 120 120 120 -t 600 -r 600 -n 1 2.4 -n 2 0.77 -n 3 0.77`

`-m 1 2 1.28 -m 2 1 1.28 -m 1 3 0.32 -m 3 1 0.32 -en 0.049975 2 0.00247491582263 -en 0.049975 3 0.000720979721217 -ej 0.05 3 2 -en 0.05 2 0.77 -en 0.087475 1 0.00622496653265 -en 0.087475 2 0.00056286521273 -ej 0.0875 2 1 -en 0.0875 1 2.4 -en 0.425 1 1.25`), with the initial frequency of the beneficial allele set to 0.02 for soft sweep simulations. **a)** ROC curves are generated as in Figure 1, with false positive rate being the fraction of neutral variants incorrectly classified as adaptive, and true positive rate being the fraction of adaptive mutations originating as a standing variant (soft sweep site) that are correctly classified as such. As might be expected, population differentiation component statistics perform well at distinguishing between neutrality and soft sweeps, while performance of iHS, XP-EHH, and ΔiHH suffers dramatically at this task relative to the task of distinguishing between neutrality and hard sweeps (inset panel). Nonetheless, SWIF(r) maintains power to detect the genomic signatures of adaptive standing variants. **b)** Although the initial frequency of the beneficial mutations is 2% in these simulations, the vast majority of these simulations result in soft sweeps in the population of interest at the time of sampling, with more than one (and in some cases many) of the original beneficial mutation origins still present at the time of sampling. In approximately 1% of our simulations, we observe a "hardening" of the sweep (i.e. only one mutation origin is present at the time of sampling). Note that we do not include any simulations in which the beneficial mutation is lost.

Supplementary Figure 31

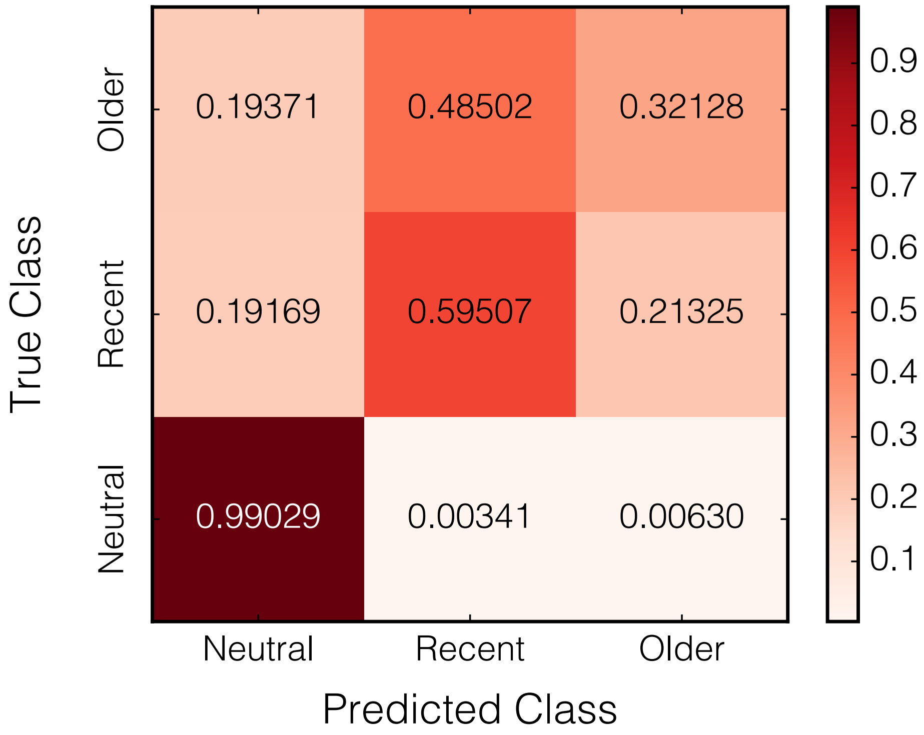

SWIF(r) extends trivially to multi-class classification. In addition to neutral simulations, SWIF(r) was trained on simulations of "recent" sweeps in the ‡Khomani San (5-30kya), and "older" sweeps (30-60kya). Training SWIF(r) on these three classes ("neutral", "recent", and "older") merely requires computing three likelihoods instead of two (see Equation 2), with all three included in the denominator of Equation 3. Posterior probabilities can then be computed for each of the three classes. We classified loci by assigning them to the class with the highest posterior probability. Values in the confusion matrix are the conditional probability of classifying a site in the predicted class, given that the site belongs to the true class. Priors are 0.9 for neutral, and 0.05 for each of the two adaptive classes. In this case, the component statistics do not carry enough power to reliably distinguish between these two sweep timings, likely because of confounding effects of sweep strength and present-day allele frequency. It is plausible that by incorporating other component statistics, SWIF(r) may be able to achieve this timing inference.

Supplementary Figure 32

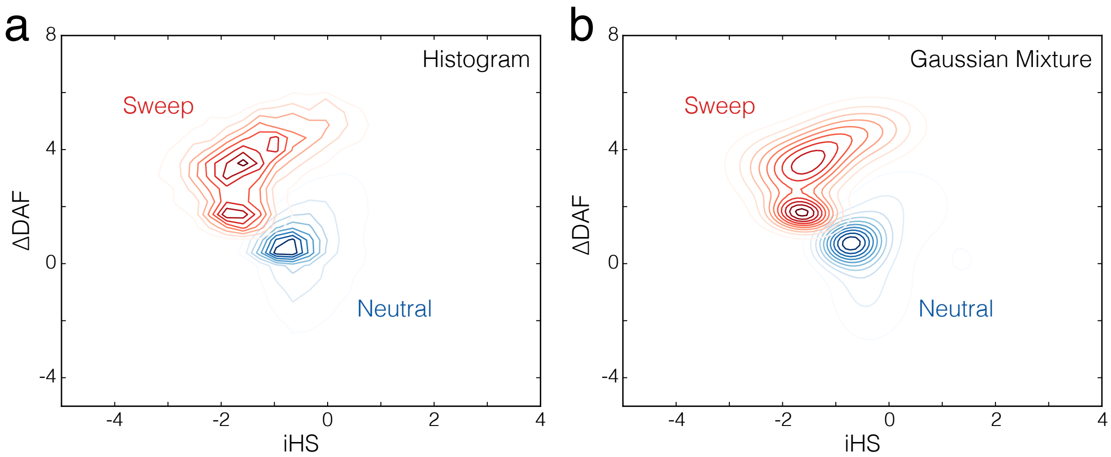

Using Gaussian mixture models to approximate joint likelihoods of selection statistics. The joint distributions of iHS and ΔDAF under neutral and sweep scenarios constructed with **a)** a 30×30 binned histogram approach, and **b)** Gaussian mixture models. The mixture models adequately smooth the distributions while maintaining their general shape and modality, thereby avoiding over-fitting.

Supplementary Figure 33

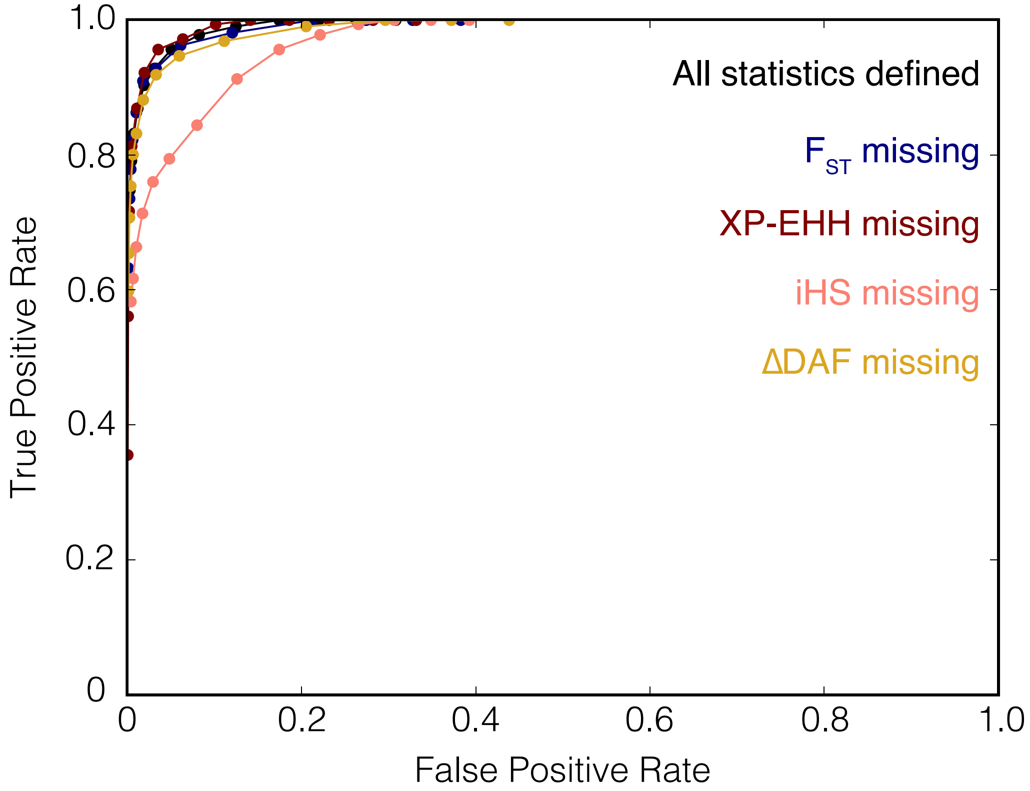

Effect of undefined statistics on classification power. In simulation, we gathered all sites with no missing statistics, and then calculated SWIF(r) sweep probabilities for the complete set of statistics, and with each statistic missing. The ROC curves show the effect of each of these missing statistics on the power of SWIF(r). XP-EHH, F_{ST}, and ΔDAF all have very little effect, but a missing value for iHS does result in lower power to distinguish adaptive mutations from neutral mutations. This differential loss of power is not surprising, since iHS is extremely useful for identifying incomplete sweeps, while XP-EHH, ΔDAF, and F_{ST} can more easily compensate for each other, as they are all most powerful for sweeps that are complete in the population of interest (Supplementary Figure 7).

Supplementary Figure 34

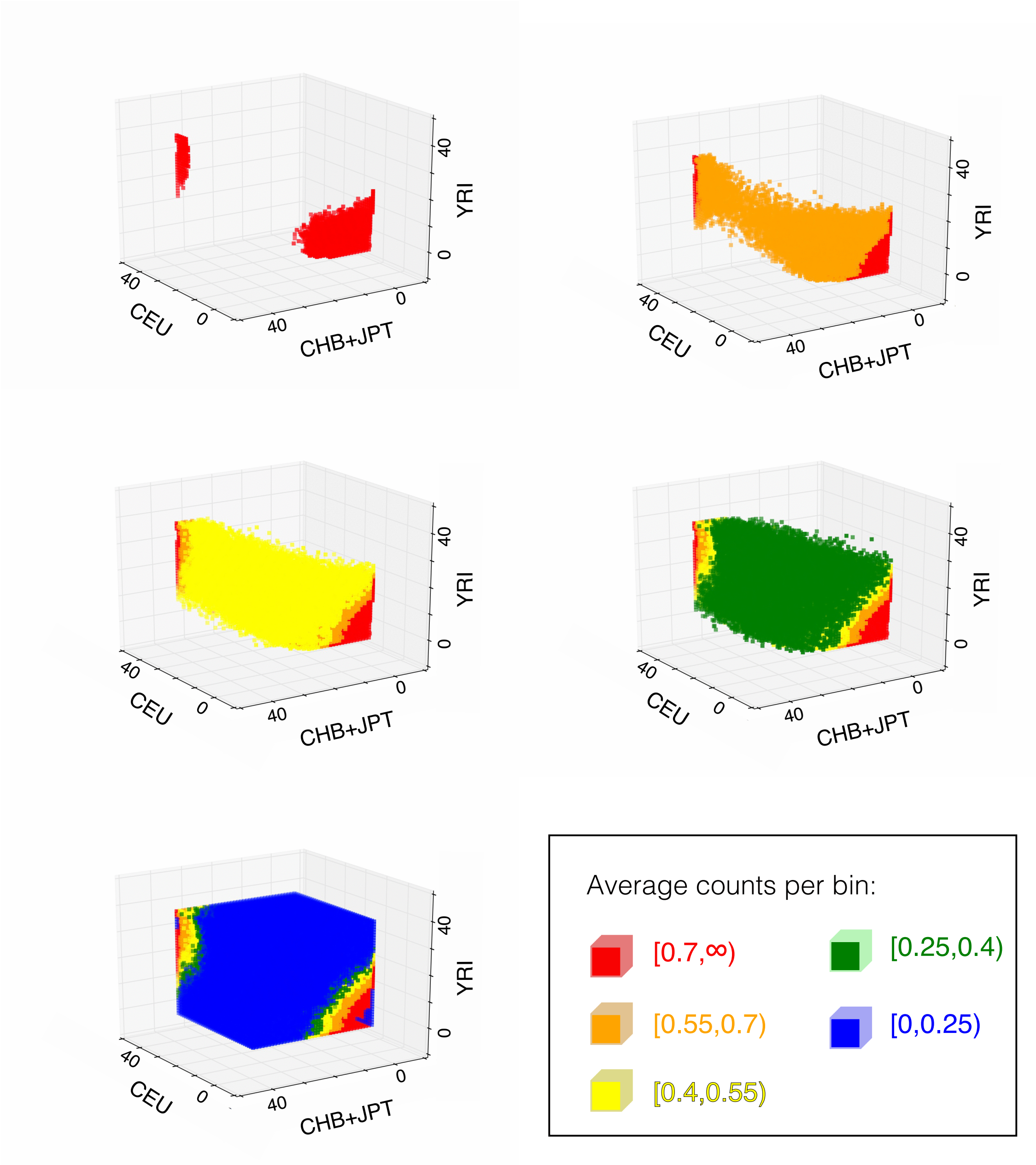

Illustration of step 2 of ascertainment modeling algorithm. For modeling ascertainment in the SNP array used to sequence the ‡Khomani San individuals in our selection scan, we defined five intervals that span the range of average count-per-bin of $40 \times 40 \times 40$ SFS space, from less than 0.25 in blue, to greater than 0.7 in red. The set of bins defined by a given color are the "SFS regions" referred to in Methods. The five plots are shown because lower-count regions tend to conceal the higher-count regions in the two-dimensional projection.

Supplementary Table 1

Undefined statistics in 1000 Genomes. Fraction of variant sites, out of 11 million, for which each of four component statistics are undefined within each of three populations.

Population	F_{ST}	XP-EHH	iHS	ΔDAF
CEU	0.5%	0.2%	77.5%	0%
CHB+JPT	0.5%	0.3%	78.9%	0%
YRI	0.5%	0.3%	67.4%	0%

Supplementary Table 2

False Discovery and Positive Predictive Value rates for SWIF(r) predictions in the 1000 Genomes dataset. Estimating empirical false discovery rates in observed genomic data requires defining a set of neutrally evolving genomic sites, which is extremely difficult to do. We use two proxies for defining these sites: first, we use "non-conserved non-coding" sites, defined as those lying at least 2kb away from annotated genes, and having a phastcons conservation score of zero, following Hernandez *et al.*[29]. We consider this to be an extremely permissive definition, as sites with low conservation scores may well be tagging nearby variants with higher scores that may or may not be present in the genotype dataset, and intergenic regions contain many regulatory and functional elements. The second proxy we use is a set of 15 putatively neutral regions comprising ∼216kb identified by Gazave *et al.*[30] using a range of criteria indicating neutrality.

For the first proxy, we calculated the false positive rate among these sites at different sweep probability thresholds, then extended this false positive rate genome-wide to obtain a prediction for the total number of false positives that would be expected in a genome scan in each population of interest (YRI, CEU, CHB+JPT). Dividing the number of predicted false positives by the total number of sites that SWIF(r) identifies above a given threshold in each population gives us an estimate of the false discovery rate (FDR), and inversely of the positive predictive value (PPV) of SWIF(r). We find in general that we predict lower FDR at higher posterior probability cutoffs, indicating that high-probability SNPs are more likely to be true signals than lower-probability SNPs. The last column contains the fraction of total predictions made by SWIF(r) that we estimate to be true, based on these values. For YRI, we only show results up to a sweep probability threshold of 0.5 because we only observe one false positive at this threshold, and we thus lack the resolution to estimate FDR or PPV for higher thresholds. Again, we note that some SNPs labeled as false positives by this scheme may nonetheless tag nearby selective sweeps, and we consider these false discovery rates to be very conservative.

Using the much stricter definition of neutrally evolving sequence developed by Gazave *et al.*[30], we see no false positives in any of the three populations, even at a sweep probability threshold of 10%.

**Population**	**Sweep Probability Threshold**	**FDR**	**PPV**	**Estimated True/Total**
YRI	0.5	0.514	0.486	7/15
CEU	0.5	0.421	0.579	138/238
	0.6	0.469	0.531	96/181
	0.7	0.459	0.541	91/168
	0.8	0.504	0.496	76/153
	0.9	0.367	0.633	80/126
	0.95	0.350	0.650	71/110
	0.98	0.339	0.661	60/91
CHB+JPT	0.5	1.00	0.00	0/342
	0.6	0.801	0.199	46/231
	0.7	0.806	0.194	39/201
	0.8	0.878	0.122	19/158
	0.9	0.461	0.539	63/117
	0.95	0.308	0.692	69/100
	0.98	0.214	0.786	72/58

Supplementary Table 3

Selection coefficients for simulations. Selection coefficients for each sweep scenario are fully determined by the start time of the sweep (all sweeps end at the time of sampling), the number of years per generation, the final allele frequency of the beneficial allele, and the effective population size for the population within which the sweep occurs. As in Schaffner *et al.*[11], N_e is 24000 for West Africa (YRI), 7700 each for Europe (CEU) and East Asia (CHB and JPT), and as in Gronau *et al.*[10], N_e is 21000 for the ‡Khomani San. The demographic model we use for the 1000 Genomes populations (YRI, CEU, CHB, JPT) assumes 20 years/generation[11], while the one we use for the ‡Khomani San study assumes 25 years/generation[10].

Start Time	Final Allele Frequency	Selective Strength (Europe, East Asia)	Selective Strength (West Africa)	Selective Strength (‡Khomani San)
5kya-present	0.2	0.033	0.0376	0.0463
	0.4	0.0369	0.0415	0.0512
	0.6	0.0402	0.0447	0.0553
	0.8	0.0441	0.0487	0.0602
	1	0.0771	0.0862	0.1065
10kya-present	0.2	0.0165	0.0188	0.0231
	0.4	0.0185	0.0207	0.0256
	0.6	0.0201	0.0224	0.0276
	0.8	0.0221	0.0243	0.0301
	1	0.0386	0.0431	0.0532
15kya-present	0.2	0.011	0.0125	0.0154
	0.4	0.0123	0.0138	0.0171
	0.6	0.0134	0.0149	0.0184
	0.8	0.0147	0.0162	0.0201
	1	0.0257	0.0287	0.0355
20kya-present	0.2	0.0083	0.0094	0.0116
	0.4	0.0092	0.0104	0.0128
	0.6	0.01	0.0112	0.0138
	0.8	0.011	0.0122	0.0150
	1	0.0193	0.0216	0.0266
25kya-present	0.2	0.0066	0.0075	0.0093
	0.4	0.0074	0.0083	0.0102
	0.6	0.008	0.0089	0.0111
	0.8	0.0088	0.0097	0.0120
	1	0.0154	0.0172	0.0213
30kya-present	0.2	0.0057	0.0065	0.0080
	0.4	0.0064	0.0072	0.0088
	0.6	0.0069	0.0077	0.0095
	0.8	0.0076	0.0084	0.0103
	1	0.0133	0.0149	0.0184
41kya-36kya	0.2			0.0462
	0.4			0.0512
	0.6			0.0553
	0.8			0.0601
	1.0			0.1064
46kya-36kya	0.2			0.0231
	0.4			0.0256
	0.6			0.0276
	0.8			0.0301
	1.0			0.0532
52kya-47kya	0.2			0.0463
	0.4			0.0512
	0.6			0.0553
	0.8			0.0602
	1.0			0.1065
57kya-47kya	0.2			0.0231
	0.4			0.0256
	0.6			0.0276
	0.8			0.0301
	1.0			0.0532

Supplementary Table 4

False Discovery and Positive Predictive Value rates for SWIF(r) predictions in the ‡Khomani dataset. Estimating empirical false discovery rates in observed genomic data requires defining a set of neutrally evolving genomic sites, which is extremely difficult to do. We use two proxies for defining these sites: first, we use "non-conserved non-coding" sites, defined as those lying at least 2kb away from annotated genes, and having a phastcons conservation score of zero, following Hernandez *et al.*[29]. We consider this to be an extremely permissive definition, as sites with low conservation scores may well be tagging nearby variants with higher scores that may or may not be present in the genotype dataset, and intergenic regions contain many regulatory and functional elements. The second proxy we use is a set of 15 putatively neutral regions comprising ~216kb identified by Gazave *et al.*[30] using a range of criteria indicating neutrality.

For the first proxy, we calculated the false positive rate among these sites at different sweep probability thresholds, then extended this false positive rate genome-wide to obtain a prediction for the total number of false positives that would be expected in a genome scan. Dividing the number of predicted false positives by the total number of sites that SWIF(r) identifies above a given threshold in the ‡Khomani San gives us an estimate of the false discovery rate (FDR), and inversely of the positive predictive value (PPV) of SWIF(r). The last column contains the fraction of total predictions made by SWIF(r) that we estimate to be true, based on these values. For YRI, we only show results up to a sweep probability threshold of 0.5 because we only observe one false positive at this threshold, and we thus lack the resolution to estimate FDR or PPV for higher thresholds. As in Supplementary Table 2, we note that some SNPs labeled as false positives by this scheme may nonetheless tag nearby selective sweeps, and we consider these false discovery rates to be very conservative.

Using the much stricter definition of neutrally evolving sequence developed by Gazave *et al.*[30], we see no false positives in any of the three populations, even at a sweep probability threshold of 10%.

**Sweep Probability Threshold**	**FDR**	**PPV**	**Estimated True/Total**
0.5	0.563	0.437	47/108
0.6	0.615	0.385	35/90
0.7	0.560	0.440	35/79
0.8	0.439	0.561	35/63
0.9	0.474	0.526	18/35
0.95	0.572	0.428	12/29
0.98	0.251	0.749	16/22

Supplementary Data 1

Selective sweep targets identified by SWIF(r) in the 1000 Genomes phase 1 dataset. Spreadsheet contains all SNPs that have a posterior sweep probability greater than 10% for each of the three populations. SNPs are identified by rsid (column A), chromosome (B), and position in genome build hg19 (C). The uncalibrated posterior sweep probability calculated by SWIF(r) is shown in column D, the calibrated probability using isotonic regression is shown in E, and the calibrated value using smoothed isotonic regression is in F (Supplementary Figure 1, Supplementary Figure 3). SNPs are annotated by gene (G), mutation type (H), and genes within 100kb (I).

Supplementary Data 2

Support for positive selection in genes identified by SWIF(r) in the 1000 Genomes phase 1 dataset. For each of the three populations (CEU, CHB+JPT, YRI), we list every gene containing a SNP with posterior sweep probability over 10% (column A), the number of such SNPs (B), and the maximum sweep probability over all such SNPs (C). The citations in the remaining columns are studies in which the gene was implicated in a positive selection scan in the population of interest. Information about these citations can be found in the last sheet of this spreadsheet.

Supplementary Data 3

Adaptive loci identified by SWIF(r) in the ‡Khomani San array dataset. Spreadsheet contains all SNPs that have posterior sweep probability greater than 10% in the ‡Khomani array dataset[25]. SNPs are identified by rsid (column A), chromosome (B), and position in hg19 (D). The derived allele frequency of the SNP in the ‡Khomani is in column C. The uncalibrated posterior sweep probability calculated by SWIF(r) is in column E, the calibrated probability using isotonic regression is shown in F, and the calibrated value using smoothed isotonic regression is in G (Supplementary Figure 2, Supplementary Figure 3). The un-calibrated posterior sweep probability is broken down into posterior probabilities for "recent" (<30kya) and "ancient" (36-47kya) in columns H and I, respectively. SNPs are also annotated by gene (J), mutation type (K), and genes within 100kb (L).

Supplementary Data 4

Exome data from 45 ‡Khomani San individuals reveals variants that have functional consequence and large allele frequency differences relative to other worldwide populations within SWIF(r)-identified genes involved in metabolism and obesity. Spreadsheet contains SNPs

of interest in exome data (Martin *et al.*[31]) within genes highlighted in Figure 3b. Each SNP is annotated with rsid and chromosome position in genome build hg19. The variant type was determined using the UCSC Genome Browser[32], and frequencies in worldwide populations were taken from phase 3 of the 1000 Genomes project[18] where available, otherwise, frequencies were taken from the Human Genome Diversity Project and/or the ExAC browser[33].

Supplementary Note 1

Adaptation of `selscan`[34] for better performance on incomplete sweeps. XP-EHH is defined as $\log \frac{\mathrm{iHH}_1}{\mathrm{iHH}_2}$, where iHH_1 and iHH_2 are "integrated haplotype homozygosities" for the population of interest and a reference population respectively. iHH is computed as the integral under the EHH curve, where EHH at a distance x from the core SNP is canonically computed as follows:

$$\mathrm{EHH} = \frac{\sum_{i=1}^{G} \binom{n_i}{2}}{\binom{N}{2}}$$

where N is the total number of chromosomes, G is the number of distinct haplotypes, and n_i is the number of chromosomes of distinct type i. In the case of incomplete sweeps from a *de novo* mutation, this definition somewhat counterintuitively leads to negative values of XP-EHH. This is due to the fact that the numerator has an upper bound of $\binom{N_a}{2} + \binom{N_A}{2}$, where N_a and N_A are the number of chromosomes that have each of the two possible alleles at the core SNP. In the reference population, there is a larger upper bound of $\binom{N}{2}$, leading to a larger value of iHH for the reference population than for the population with the adaptive mutation.

Following Wagh *et al.*[35], we instead define EHH as follows:

$$EHH = \frac{\sum_{i=1}^{G} \binom{n_i}{2}}{\binom{N_a}{2} + \binom{N_A}{2}}$$

When XP-EHH is defined this way, it is far more powerful for detecting sweeps, and does not return negative values for incomplete sweeps. We modified `selscan`'s source code to implement this change. These modifications can be found at `https://github.com/lasugden/selscan` (original software at `https://github.com/szpiech/selscan`).

Supplementary Note 2

Removal of ΔiHH from analyses of real data. While other statistics are distributed approximately normally after normalization, ΔiHH maintains a very long tail (1-2 orders of magnitude longer than the tails of other statistics), which is exacerbated by normalization. This problem is compounded by the fact that

ΔiHH is an absolute value, leading to high scores even when the ancestral haplotype is longer than the derived haplotype. In Supplementary Figure 5, we show the distribution of ΔiHH and iHS values for sites with SWIF(r) sweep probability $\geq 50\%$ in the 1000 Genomes dataset using SWIF(r) with ΔiHH included as a component statistic. Note the scale of both statistics; while iHS ranges from -4 to 4, ΔiHH ranges from 0 to 100 (in simulations, the probability mass for ΔiHH lies mostly below 5, and entirely below 14). The plot shows that ΔiHH can be extremely high, even for values of iHS that are positive and thus provide evidence against selective sweeps. Furthermore, the more negative iHS values correspond to more moderate ΔiHH values, and not the most extreme ones, thus leading to a large number of false positives. For these reasons, we removed ΔiHH as a component statistic for analysis of simulations and genotype data from the 1000 Genomes and the ‡Khomani San.

Supplementary Note 3

Processing of 1000 Genomes Data. We performed a genome-wide scan for selective sweeps in four populations (YRI, CEU, CHB, JPT) using phase 1 of the 1000 Genomes Project (May 2011 release), with CHB and JPT grouped together and representing East Asia. We filtered the samples to omit children from parent-child pairs and trios (NA07048, NA10847, and NA10851 from CEU and NA19129 from YRI), and we only analyzed single-nucleotide variants. We also removed loci that were monomorphic within the filtered set of unrelated individuals across the four populations analyzed. We used ancestral allele information provided by the 1000 Genomes Project.

Supplementary Note 4

Calculation of migration rates from YRI and CEU to ‡Khomani San. Uren *et al.*[25] use the software package Tracts[36] to infer the magnitude of genetic contributions to present-day ‡Khomani San individuals from three source populations: KhoeSan (data presented in Uren *et al.*[25] and Schuster *et al.*[37]), LWK (1000 Genomes), and CEU (1000 Genomes). In our simulations, we use YRI as a proxy population for LWK (i.e. migration rates learned in Uren *et al.* from LWK are implemented in our demographic model as migration rates from YRI), as both are Bantu-speaking populations, which all originate from west central Africa. Therefore both populations provide an appropriate source for the Bantu ancestry in the ‡Khomani San. The migration rates reported by the Tracts software represent the proportion of the target population that is replaced at a given generation by a source population, starting in this case 14 generations ago. Prior to 14 generations ago, we assume that the ‡Khomani San population is made up of 100% KhoeSan ancestry, and we converted the Tracts migration rates into migration rates from only two source populations (YRI and

CEU) into the third (‡Khomani San). We achieved this by going through an intermediate step in which we calculated a matrix of ancestry proportions at each generation. The table below shows the tracts output from Uren *et al.*[25] (migration rates m_S, m_Y, and m_C at each generation from 14 generations ago to present. We then calculate the ancestry proportions at each generation, initializing at generation 14 (m_Y and m_S at generation 14 add to 1, indicating that the ancestry proportions at this time point are equal to the migration rates), and working backwards. If we define m_S^i, m_Y^i, m_C^i to be the migration rates at generation i in this 3-source population model, and a_S^i, a_Y^i, a_C^i to be the ancestry proportions at generation i, then we can calculate these by the following recursive formulas, where $(1 - m_S^i - m_Y^i - m_C^i)$ represents the fraction of the population not being replaced at the given generation.

$$\begin{aligned} a_S^i &= (1 - m_S^i - m_Y^i - m_C^i)a_S^{i+1} + m_S^i \\ a_Y^i &= (1 - m_S^i - m_Y^i - m_C^i)a_Y^{i+1} + m_Y^i \\ a_C^i &= (1 - m_S^i - m_Y^i - m_C^i)a_C^{i+1} + m_C^i \end{aligned}$$

The entries in the following table for a_S, a_Y, and a_C are calculated using these formulas. Finally, we need to convert these ancestry proportions into migration rates for a model with two population sources (YRI and CEU). For simplicity, we assume a one-generation migration pulse from YRI at generation 14, and a one-generation migration pulse from CEU at generation 7. We note that in order to achieve a present-day CEU ancestry proportion of 0.179, the CEU migration rate must be 0.179 (the migration rate is again defined as the fraction of the population being replaced by the source population). Finally, to achieve a present-day YRI ancestry proportion of 0.186, the migration rate from YRI must be 0.227, since $(1 - 0.179) \times 0.227 = 0.186$ (this accounts for the proportion of YRI ancestry that gets replaced by CEU ancestry in generation 7).

Supplementary Note 5

Generation of exome and array data in San population. ‡Khomani San individuals were sampled in 2006 in Upington, South Africa and neighboring villages. Institution Review Board (IRB) approval for assessment of genetic diversity and ancestry inference was obtained from Stanford University [Protocol 13829] and Stony Brook University [Protocol 727494-5]. Still-living individuals were re-consented in 2011 (IRB approved from Stanford University and Stellenbosch University, South Africa). ‡Khomani N|u-speaking individuals, local community leaders, traditional leaders, non-profit organizations and a legal counselor were all consulted about the project aims before DNA collection commenced. All individuals initially orally

generation	m_S	m_Y	m_C	a_S	a_Y	a_C
1	0	0	0	0.635	0.186	0.179
2	0	0	0	0.635	0.186	0.179
3	0	0	0	0.635	0.186	0.179
4	0.240	0.031	0	0.635	0.186	0.179
5	0.052	0.005	0	0.542	0.212	0.246
6	0	0	0.151	0.520	0.219	0.261
7	0	0	0.130	0.612	0.258	0.130
8	0	0	0	0.703	0.297	0
9	0	0	0	0.703	0.297	0
10	0	0	0	0.703	0.297	0
11	0	0	0	0.703	0.297	0
12	0	0	0	0.703	0.297	0
13	0.527	0.222	0	0.703	0.297	0
14	0.703	0.297	0	0.703	0.297	0

consented to participate in the project in the presence of a witness fluent in the native language, and were re-consented with written consent. DNA was collected via saliva (Oragene kits). Ancestry and genotyping details of individuals included here can be found in Uren *et al.*[25].

90 KhoeSan DNA samples were captured with 3 exome platforms: 74 samples on an Agilent SureSelect Human All Exon V2 44Mb, 8 samples on an Agilent SureSelect Human All Exon 50Mb, and 8 samples on an Agilent SureSelect Human All Exon V4+UTRs 71Mb (Martin *et al.*[31]). Illumina short-read sequencing data were jointly processed according to the best practice pipeline of the 1000 Genomes Project[18]. Reads were aligned to the hg19 reference genome using bwa-mem 0.7.10[38]. The resultant BAM files were then sorted and marked for duplicate reads using the Picard v1.92 toolkit (`http://broadinstitute.github.io/picard/`). The following programs were then run with GATKv3.2.2[39]: RealignerTargetCreator, IndelRealigner, BaseRecalibrator, PrintReads, HaplotypeCaller, GenotypeGVCFs, and VariantRecalibrator, and ApplyRecalibration. During the HaplotypeCaller step, we filtered reads to include only the Agilent capture regions ± 100 bp of padding.

For phasing, exome data were merged with Illumina SNP arrays for each of 87 individuals to improve accuracy by providing a broader SNP scaffold. After merging and filtering to 5% genotyping missingness using vcftools, 759,586 SNPs remained. Data were phased using a two-step phasing process as follows: first, 25 related individuals, consisting of 3 trios and 8 duos, were used to create a family reference panel; after phasing unrelated individuals using default protocols in SHAPEIT2[40], pedigree information was used via duo-trio phasing with duoHMM[41] to inform the phasing of the unrelated individuals in a second step, improving phasing accuracy overall by correcting phase switch errors.

‡Khomani San individuals were genotyped on two SNP array platforms: the Illumina OmniExpress and OmniExpressPlus chips. Only SNPs shared between these two platforms[25] were retained for investigation of

adaptive sites, in order to avoid allele frequency biases related to platform choice; there is broad overlap between these arrays, with the OmniExpressPlus SNP array containing an additional 250k sites. 86 individuals were genotyped and were phased using only pedigree information. This pedigree-phased dataset provides the highest possible SNP density for these individuals and, after quality control filtering, included just under 650k SNPs.

For the analyses presented here, we selected 45 unrelated ‡Khomani San individuals with low rates of recent European and Bantu-derived admixture from the phased datasets. These individuals were identified by running ADMIXTURE v1.2[42] on a joint dataset of Illumina SNP arrays consisting of diverse populations of individuals[25] and selecting those KhoeSan individuals with >90% Khoesan ancestry at $K = 6$. Six was determined to be the K value that best fit the data, using both cross-validation procedures and cluster appearance.

Supplementary Note 6

SWIF(r) signals associated with muscle-based phenotypes. Both *MYH15* and *TTN* have been associated with obesity and metabolism phenotypes (Table 1). Additionally, both genes encode striated muscle. *MYH15* has been associated with coronary heart disease[43], and *TTN* mutations have been associated with cardiomyopathy[44], with RNAseq data indicating that expression is highest in heart tissue[45]. Associations for *MYH15* and *TTN* with the obesity and metabolism phenotype may be a consequence of these other functions and associations. Exome support for selection acting within these genes is given below; allele frequencies for populations other than the ‡Khomani San refer to frequencies found in the 1000 Genomes phase 3 dataset[18].

TTN (titin): We find 6 missense mutations within 50kb of the SWIF(r) signal; in itself, this may not be unusual given the exon richness in this gene. However, many of the mutations have a population frequency of approximately 50% in the Khomani San, while being absent or having much lower frequencies in other worldwide populations. For example, the derived asparagine to isoleucine (rs11900987) mutation is conserved amongst mammals[32], <1% in other human populations, but present at 48% in our San sample. Other mutations segregating at similar frequency within 50kb suggest that a high frequency haplotype is under adaptive evolution the ‡Khomani San.

MYH15 (myosin heavy chain 15): Our exome analysis found two missense mutations in *MYH15* with large allele frequency deviations. the G allele of rs9868484, which lies ∼4kb from the SWIF(r) signal, is at a frequency of 71% in our sample, relative to a maximum frequency of 40% elsewhere in Africa. The T allele of

rs1078456, $\sim$50kb from the SWIF(r) signal, is at a frequency of 22%, relative to a maximum of 4% worldwide. In addition, a splice region variant $\sim$38kb from the SWIF(r) signal, rs113330737, has derived allele frequency 46% in the ‡Khomani sample relative to a maximum of 1% worldwide.

Supplementary Note 7

`cosi` demographic parameter file for 1000 Genomes dataset

```
length 1000000
mutation_rate 1.5e-8
recomb_file <filename>
gene_conversion_rate 4.5e-9

pop_define 1 european
pop_define 2 asian
pop_define 3 african

#initial sizes and sample sizes
pop_size 1 7700
sample_size 1 120
pop_size 2 7700
sample_size 2 120
pop_size 3 24000
sample_size 3 120

#Migration start
pop_event migration_rate ”afr to eur migration” 3 1 1505 .000032
pop_event migration_rate ”eur to afr migration” 1 3 1504 .000032
pop_event migration_rate ”afr to as migration” 3 2 1503 .000008
pop_event migration_rate ”as to afr migration” 2 3 1502 .000008

#Migration end
pop_event migration_rate ”afr to eur migration” 3 1 1996 0
pop_event migration_rate ”eur to afr migration” 1 3 1995 0
```

```
pop_event migration_rate "afr to as migration" 3 2 1994 0
pop_event migration_rate "as to afr migration" 2 3 1993 0

#Recent Bottlenecks:
pop_event bottleneck "african bottleneck" 3 1997 .008
pop_event bottleneck "asian bottleneck" 2 1998 .067
pop_event bottleneck "european bottleneck" 1 1999 .02

#Population splits:
pop_event split "asian and european split" 1 2 2000
pop_event split "out of Africa" 3 1 3500

#Out-of-africa bottleneck
pop_event bottleneck "OoA bottleneck" 1 3499 .085

#Ancestral expansion
pop_event change_size "african pop size" 3 17000 12500
```

cosi demographic parameter file for ‡Khomani dataset

```
length 1000000
mutation_rate 1.5e-8
recomb_file <filename>
gene_conversion_rate 4.5e-9

pop_define 1 european #E
pop_define 2 han_chinese #H
pop_define 3 yoruban #Y
pop_define 4 khomani #K

#initial sizes and sample sizes
pop_size 1 9700
sample_size 1 164
pop_size 2 5800
```

```
sample_size 2 372
pop_size 3 17800
sample_size 3 174
pop_size 4 21000
sample_size 4 90

#population size changes
pop_event change_size ”H” 3 1240 3500
pop_event change_size ”HE” 2 1441 1200
pop_event change_size ”HEY” 3 1881 11500
pop_event change_size ”HEYK” 4 5241 8700

#migration to Khomani
pop_event migration_rate ”YRI to Khomani migration” 3 4 14 0.227
pop_event migration_rate ”YRI to Khomani migration end” 3 4 15 0
pop_event migration_rate ”CEU to Khomani migration” 1 4 6 0.179
pop_event migration_rate ”CEU to Khomani migration end” 1 4 7 0

#population splits:
pop_event split ”CE” 2 1 1440
pop_event split ”CEY” 3 2 1880
pop_event split ”CEYK” 4 3 5240
```

Supplementary Note 8

Selection Statistic Calculations. For each segregating site within the neutral simulations, and for the adaptive site in sweep simulations, we computed component selection statistics (F_{ST}, XP-EHH, iHS, ΔiHH, ΔDAF) with respect to a population of interest (the population undergoing a sweep in sweep simulations, and a population chosen uniformly at random in the case of neutral simulations), ignoring sites for which the derived allele frequency in the population of interest was zero. F_{ST} for each of the pairwise population comparisons involving the population of interest was computed as in Weir *et al.*[46], and then averaged together. iHS was computed with `selscan`[34]. ΔiHH was calculated as defined in Grossman *et al.*[17]: ΔiHH$|$iHH$_{\text{ancestral}}$-iHH$_{\text{derived}}|$, where iHH is the integrated haplotype homozygosity defined in Voight *et al.*[4].

ΔDAF was also calculated as defined in Grossman *et al.*[17]: $\Delta\text{DAF} = \text{DAF}_1 - \frac{1}{2}(\text{DAF}_2 + \text{DAF}_3)$ where DAF_1 is the derived allele frequency in the population of interest, and DAF_2 and DAF_3 are the derived allele frequencies in the other two populations. XP-EHH was computed with a minor alteration of `selscan` in which EHH is computed as in Wagh *et al.*[35] (see "Adaptation of `selscan` for better performance on incomplete sweeps"), and XP-EHH values were also normalized with population-specific mean and standard deviation, learned from neutral simulations, to correct for inherent biases based on linkage disequilibrium structure.

Small adjustments were required for computing these component statistics using the ‡Khomani San simulations and genotype array data[25]. In these analyses, there are three outgroups (western Africa, Europe, and eastern Asia) instead of two: XP-EHH was defined as the maximum XP-EHH value across the three comparisons; and ΔDAF was defined as
$\text{DAF}_{\ddagger\text{Khomani San}} - \frac{1}{3}(\text{DAF}_{\text{European}} + \text{DAF}_{\text{East Asian}} + \text{DAF}_{\text{West African}})$. ΔiHH was not included in these analyses (see "Removal of ΔiHH from analyses of real data").

Results for each component statistic were normalized within 1MB regions, with iHS and ΔiHH being normalized first within frequency bins as in Voight *et al.*[4]. For the CMS, we learned one-dimensional probability distributions for each (scenario, component statistic) pair in 60 evenly spaced bins, with minimum and maximum values below, chosen to encompass the full range of values observed across all neutral and sweep simulations:

component statistic	minimum	maximum
mean F_{ST}	-1	9
maximum XP-EHH	-3	10
iHS	-5	4
ΔiHH	-3	10
ΔDAF	-5	8

When computing statistics for either 1000 Genomes or ‡Khomani San genotype data, component statistics were normalized genome-wide, following Grossman *et al.*[16].

References

1. Murphy, A. H. & Winkler, R. L. Reliability of subjective probability forecasts of precipitation and temperature. *Appl Stat* 41–47 (1977).

2. Niculescu-Mizil, A. & Caruana, R. Predicting good probabilities with supervised learning. In *Proceedings of the 22nd international conference on Machine learning*, 625–632 (ACM, 2005).

3. Nielsen, R. *et al.* Genomic scans for selective sweeps using SNP data. *Genome Res* **15**, 1566–1575 (2005).

4. Voight, B. F., Kudaravalli, S., Wen, X. & Pritchard, J. K. A Map of Recent Positive Selection in the Human Genome. *PLoS Biol* **4**, e72 (2006).

5. Lin, K., Li, H., Schlötterer, C. & Futschik, A. Distinguishing positive selection from neutral evolution: boosting the performance of summary statistics. *Genetics* **187**, 229–44 (2011).

6. Sheehan, S. & Song, Y. S. Deep learning for population genetic inference. *PLoS Comput Biol* **12**, e1004845 (2016).

7. Garud, N. R., Messer, P. W., Buzbas, E. O. & Petrov, D. A. Recent selective sweeps in North American Drosophila melanogaster show signatures of soft sweeps. *PLoS Genet* **11**, e1005004 (2015).

8. Tajima, F. Statistical method for testing the neutral mutation hypothesis by DNA polymorphism. *Genetics* **123**, 585–595 (1989).

9. Du, P. & Tang, L. Transformation-invariant and nonparametric monotone smooth estimation of ROC curves. *Stat Med* **28**, 349–359 (2009).

10. Gronau, I., Hubisz, M. J., Gulko, B., Danko, C. G. & Siepel, A. Bayesian inference of ancient human demography from individual genome sequences. *Nat Genet* **43**, 1031–1034 (2011).

11. Schaffner, S. *et al.* Calibrating a coalescent simulation of human genome sequence variation. *Genome Res* **15**, 1576–83 (2005).

12. Gravel, S. *et al.* Demographic history and rare allele sharing among human populations. *Proc Natl Acad Sci USA* **108**, 11983–11988 (2011).

13. Messer, P. W. SLiM: simulating evolution with selection and linkage. *Genetics* **194**, 1037–1039 (2013).

14. Messer, P. W. & Petrov, D. A. Frequent adaptation and the McDonald–Kreitman test. *Proc Natl Acad Sci USA* **110**, 8615–8620 (2013).

15. Enard, D., Messer, P. W. & Petrov, D. A. Genome-wide signals of positive selection in human evolution. *Genome Res* **24**, 885–895 (2014).

16. Grossman, S. R. *et al.* Identifying recent adaptations in large-scale genomic data. *Cell* **152**, 703–713 (2013).

17. Grossman, S. *et al.* A composite of multiple signals distinguishes causal variants in regions of positive selection. *Science* **327**, 883–6 (2010).

18. 1000 Genomes Project Consortium *et al.* A global reference for human genetic variation. *Nature* **526**, 68–74 (2015).

19. Atkinson, A., Garnier, S., Afridi, S., Fumoux, F. & Rihet, P. Genetic variations in genes involved in heparan sulphate biosynthesis are associated with Plasmodium falciparum parasitaemia: a familial study in Burkina Faso. *Malaria journal* **11**, 108 (2012).

20. Bersaglieri, T. *et al.* Genetic signatures of strong recent positive selection at the lactase gene. *Am J Hum Genet* **74**, 1111–1120 (2004).

21. Visser, M., Palstra, R.-J. & Kayser, M. Human skin color is influenced by an intergenic DNA polymorphism regulating transcription of the nearby BNC2 pigmentation gene. *Hum Mol Genet* ddu289 (2014).

22. Lao, O., De Gruijter, J., van Duijn, K., Navarro, A. & Kayser, M. Signatures of positive selection in genes associated with human skin pigmentation as revealed from analyses of single nucleotide polymorphisms. *Ann Hum Genet* **71**, 354–369 (2007).

23. Pybus, M. *et al.* Hierarchical boosting: a machine-learning framework to detect and classify hard selective sweeps in human populations. *Bioinformatics* btv493 (2015).

24. McEvoy, B. P., Powell, J. E., Goddard, M. E. & Visscher, P. M. Human population dispersal "Out of Africa" estimated from linkage disequilibrium and allele frequencies of SNPs. *Genome Res* **21**, 821–829 (2011).

25. Uren, C. *et al.* Fine-scale human population structure in southern Africa reflects ecogeographic boundaries. *Genetics* **204**, 303–314 (2016).

26. Bandelt, H.-J., Forster, P. & Röhl, A. Median-joining networks for inferring intraspecific phylogenies. *Mol Biol Evol* **16**, 37–48 (1999).

27. Akbari, A. *et al.* Fine-mapping the Favored Mutation in a Positive Selective Sweep. *bioRxiv* 139055 (2017).

28. Ewing, G. & Hermisson, J. MSMS: a coalescent simulation program including recombination, demographic structure and selection at a single locus. *Bioinformatics* **26**, 2064–2065 (2010).

29. Hernandez, R. D. *et al.* Classic selective sweeps were rare in recent human evolution. *Science* **331**, 920–924 (2011).

30. Gazave, E. *et al.* Neutral genomic regions refine models of recent rapid human population growth. *Proc Natl Acad Sci USA* **111**, 757–762 (2014).

31. Martin, A. R. *et al.* An unexpectedly complex architecture for skin pigmentation in africans. *Cell* **171**, 1340–1353 (2017).

32. Kent, W. J. *et al.* The human genome browser at UCSC. *Genome Res* **12**, 996–1006 (2002).

33. Lek, M. *et al.* Analysis of protein-coding genetic variation in 60,706 humans. *Nature* **536**, 285–291 (2016).

34. Szpiech, Z. A. & Hernandez, R. D. selscan: an efficient multi-threaded program to perform EHH-based scans for positive selection. *Mol Biol Evol* msu211 (2014).

35. Wagh, K. *et al.* Lactase persistence and lipid pathway selection in the Maasai. *PLoS One* **7**, e44751 (2012).

36. Gravel, S. Population genetics models of local ancestry. *Genetics* **191**, 607–619 (2012).

37. Schuster, S. C. *et al.* Complete Khoisan and Bantu genomes from southern Africa. *Nature* **463**, 943 (2010).

38. Li, H. Aligning sequence reads, clone sequences and assembly contigs with BWA-MEM. *arXiv preprint arXiv:1303.3997* (2013).

39. McKenna, A. *et al.* The Genome Analysis Toolkit: a MapReduce framework for analyzing next-generation DNA sequencing data. *Genome research* **20**, 1297–1303 (2010).

40. Delaneau, O., Zagury, J.-F. & Marchini, J. Improved whole-chromosome phasing for disease and population genetic studies. *Nat Methods* **10**, 5–6 (2013).

41. O'Connell, J. *et al.* A general approach for haplotype phasing across the full spectrum of relatedness. *PLoS Genet* **10**, e1004234 (2014).

42. Alexander, D. H., Novembre, J. & Lange, K. Fast model-based estimation of ancestry in unrelated individuals. *Genome Res* **19**, 1655–1664 (2009).

43. Bare, L. A. *et al.* Five common gene variants identify elevated genetic risk for coronary heart disease. *Genet Med* **9**, 682–689 (2007).

44. Begay, R. L. *et al.* Role of titin missense variants in dilated cardiomyopathy. *J Am Heart Assoc* **4**, e002645 (2015).

45. Lonsdale, J. *et al.* The genotype-tissue expression (GTEx) project. *Nat Genet* **45**, 580–585 (2013).

46. Weir, B. S. Genetic Data Analysis II. *Biometrics* **53**, 392 (1997).
