# Supplementary material for: Localization of adaptive variants in human genomes using averaged one-dependence estimation
Source: Nat Commun. 2018 Feb 19;9:703. doi: 10.1038/s41467-018-03100-7 (PMC5818606; doi:10.1038/s41467-018-03100-7)
Supplement: Supplementary file 3 — Description of Additional Supplementary Files [file 41467_2018_3100_MOESM3_ESM.pdf]

Description of Additional Supplementary Files

File Name: Supplementary Data 1
Description: Selective sweep targets identified by SWIF(r) in the 1000 Genomes phase 1 dataset Spreadsheet contains all SNPs that have a posterior sweep probability greater than 10% for each of the three populations. SNPs are identified by rsid (column A), chromosome (B), and position in genome build hg19 (C). The uncalibrated posterior sweep probability calculated by SWIF(r) is shown in column D, the calibrated probability using isotonic regression is shown in E, and the calibrated value using smoothed isotonic regression is in F (Supplementary Figure 1, Supplementary Figure 3). SNPs are annotated by gene (G), mutation type (H), and genes within 100kb (I).

File Name: Supplementary Data 2
Description: Support for positive selection in genes identified by SWIF(r) in the 1000 Genomes phase 1 dataset For each of the three populations (CEU, CHB+JPT, YRI), we list every gene containing a SNP with posterior sweep probability over 10% (column A), the number of such SNPs (B), and the maximum sweep probability over all such SNPs (C). The citations in the remaining columns are studies in which the gene was implicated in a positive selection scan in the population of interest. Information about these citations can be found in the last sheet of this spreadsheet.

File Name: Supplementary Data 3
Description: Adaptive loci identified by SWIF(r) in the ‡ Khomani San array dataset Spreadsheet contains all SNPs that have posterior sweep probability greater than 10% in the ‡K homani array dataset. SNPs are identified by rsid (column A), chromosome (B), and position in hg19 (D). The derived allele frequency of the SNP in the ‡ Khomani is in column C. The uncalibrated posterior sweep probability calculated by SWIF(r) is in column E, the calibrated probability using isotonic regression is shown in F, and the calibrated value using smoothed isotonic regression is in G (Supplementary Figure 2, Supplementary Figure 3). The un-calibrated posterior sweep probability is broken down into posterior probabilities for “recent” (<30kya) and “ancient” (36-47kya) in columns H and I, respectively. SNPs are also annotated by gene (J), mutation type (K), and genes within 100kb (L).

File Name: Supplementary Data 4
Description: Exome data from 45 ‡Khomani San individuals reveals variants that have functional consequence and large allele frequency differences relative to other worldwide populations within SWIF(r)-identified genes involved in metabolism and obesity Spreadsheet contains SNPs of interest in exome data within genes highlighted in Figure 3B. Each SNP is annotated with rsid and chromosome position in genome build hg19. The variant type was determined using the UCSC Genome Browser, and frequencies in worldwide populations were taken from phase 3 of the 1000 Genomes project where available, otherwise, frequencies were

taken from the Human Genome Diversity Project and/or the ExAC browser.
